# Effects of Insecticide Stress on Expression of *NlABCG* Transporter Gene in the Brown Planthopper, *Nilaparvata lugens*

**DOI:** 10.3390/insects10100334

**Published:** 2019-10-08

**Authors:** Hong Yang, Cao Zhou, Xi-Bin Yang, Gui-Yun Long, Dao-Chao Jin

**Affiliations:** 1 Provincial Key Laboratory for Agricultural Pest Management of Mountainous Regions, Institute of Entomology, Guizhou University, Guiyang 550025, China; axyridis@163.com (H.Y.); zhoucao2011@163.com (C.Z.); yangxibingz@126.com (X.-B.Y.); 15180860256@163.com (G.-Y.L.); 2 College of Tobacco Science, Guizhou University, Guiyang 550025, China

**Keywords:** *Nilaparvata lugens*, ATP-binding cassette transporter, thiamethoxam, abamectin, cyantraniliprole, response mechanism

## Abstract

The brown planthopper (BPH), *Nilaparvata lugens*, is an important pest of rice that severely affects production. Insecticides are an important means of controlling BPH, but their long-term use has led to resistance. To provide insight into BPH responses to insecticide stress, we determined the expression levels of BPH *ABCG* transporter genes under treatment with thiamethoxam, abamectin, and cyantraniliprole at LC_10_, LC_25_, LC_50_, and LC_90_. We cloned 13 BPH *ABCG* transporters, named *NlABCG1* to *NlABCG13*. Conservative domain analysis showed that all 13 transporters have one nucleotide binding domain and one transmembrane domain, typical of semi-molecular transporters. Real-time quantitative PCR showed that thiamethoxam, abamectin, and cyantraniliprole stress increased the expression of some *NlABCG* transporters gene in BPH. However, after treatment with thiamethoxam at LC_25_ and abamectin at LC_10_, there was no significant upregulation of *NlABCG*. These results indicate that the expression of *NlABCG* varies in response to stress from different insecticides. These findings provide baseline information for further understanding of the molecular mechanisms of insecticide resistance in BPH.

## 1. Introduction

The brown planthopper (BPH), *Nilaparvata lugens*, an important migratory pest of rice in Asia that consumes the phloem of the rice plant and lays eggs on the leaf sheath, causing a decline in yield. It also spreads viral diseases such as grass stunt and ragged stunt [1,2,3]. BPH is a typical r-strategic pest with a very high intrinsic growth rate and strong adaptability to the environment. Conventionally, chemical control has been used to control BPH [4,5], but resistance to organophosphates, carbamates, pyrethroids, neonicotinoids, insect growth regulators, and phenylpyrazoles [3,6,7,8] has been observed, thereby hindering its control [9].

The ATP-binding cassette (ABC) transporter family includes various transporters present across organisms from bacteria to humans [10,11] and transport inorganic ions, sugars, amino acids, lipids, lipopolysaccharides, polypeptides, metals, xenobiotics, and chemotherapeutic drugs [12]. This group can be subdivided into eight major subfamilies (A-H) in insects [13]. Studies on ABC transporters in eukaryotes have revealed that some such transporters are capable of transporting structurally unrelated compounds [14,15], while studies on ABC transporters in insects have focused on their transport of exogenous substances and their involvement in insecticide resistance. For example, in vitro experiments with *Helicoverpa armigera* showed that the presence of P-glycoprotein might be one of the reasons for insecticide resistance in this insect [16]. Studies on *Drosophila melanogaster* using the CRISPR-Cas9 system indicate that Mdr65 knockouts were more susceptible to all neuroactive insecticides tested [17]. In addition, the ABC transporter G subfamily gene *(ABCG*) in *Sogatella furcifera* is involved in the adaptation to insecticide stress [18,19]. Eight ABC transporters in the *ABCB/C/D/G* subfamily may be involved in the resistance of *Laodelphax striatellus* to chlorpyrifos, deltamethrin, and imidacloprid [20], and the *ABCB*, *ABCC*, while the *ABCG* subfamily genes are highly expressed in a pyrethroid-resistant strain of *Aedes aegypti* [21]. These studies suggest that the presence or upregulated expression of ABC transporters is directly related to insect resistance.

ABC transporters have been systematically studied in many insects [20,21,22,23,24,25,26,27,28,29,30]. However, the effects of insecticides on ABC transporters in BPH are not fully understood. In this study, we aimed to demonstrate the role of *ABCG* transporter genes in the response to insecticide stress, and to lay a foundation for understanding the molecular mechanism of resistance of BPH.

## 2. Materials and Methods

### 2.1. Insects and Insecticides

*Nilaparvata lugens* individuals were collected from a rice field in Huaxi, Guiyang, Guizhou, China (26°31′302″ N, 106°62′294″ E) in 2013 and maintained in a laboratory on rice (TN1) seedlings at a temperature of 25 ± 1 °C and relative humidity of 70 ± 10% under a 16:8 h (L:D) photoperiod without exposure to insecticides. Third instar nymphs were used in the study. Thiamethoxam (96%, technical formulation) was obtained from PFchem Co., Ltd. (Nanjing, Jiangsu, China), abamectin (96.4%, technical formulation) was obtained from Shandong Qilu King-Phar Pharmaceutical Co., Ltd. (Jinan, Shandong, China), and cyantraniliprole (98%, technical formulation) was obtained from Guangxi Pingle Pesticide Factory (Guilin, Guangxi, China).

### 2.2. Bioassay to Determine Insecticide Concentrations for Treatment

A bioassay to identify the LC_10_-LC_90_ (resulting in 10% - 25% mortality) for each insecticide was carried out using third instar nymphs according to a previously reported rice stem dipping method [31,32]. Thiamethoxam, abamectin, and cyantraniliprole were dissolved in acetone and diluted to the required concentrations with 0.1% Triton X-100, respectively, and 0.1% Triton X-100 was also used as a blank control. Rice plants at the tillering stage were selected, washed, cut into stems of approximately 25 cm in length, and dried in the shade. These were soaked in the experimental insecticide solutions for 30 s, then dried and placed in glass tubes (300 mm height × 30 mm diameter) placed horizontally and open at both ends. Twenty healthy nymphs of similar size were selected and placed in the glass tubes, which were then placed upright after all nymphs climbed onto the rice stems. The experimental conditions were as follows: temperature 25 ± 1 °C, relative humidity 70 ± 10%, and illumination 16:8 in an artificial climate chamber for 48 h. At this point, the number of dead insects was counted. A toxicological regression equation was fitted using the Probit model in SPSS 22.0, and LC_10_, LC_25_, LC_50_, and LC_90_ were calculated.

### 2.3. Insecticide Stress at Known Concentrations

Following quantitation of LC_10_, LC_25_, LC_50_, and LC_90_ for thiamethoxam, abamectin, and cyantraniliprole, these concentrations were used in a similar format to induce insecticide stress in a further 300 nymphs using the same rice stem dipping method. As before, 0.1% Triton X-100 aqueous solution was used as a blank control. Rearing conditions were as described in the previous section. After 48 h, 15 nymphs were taken from each sample and stored at −80 °C. Each treatment was repeated three times.

### 2.4. Total RNA Extraction and cDNA Synthesis

Reserved nymphs were placed in a grinding tube and disrupted using Precellys^®^ 24 lysis/homogenizer (Bertin Technologies, Montigny le Bretonneux, France). Total RNA was extracted using E.Z.N.A.^®^ HP Total RNA Kit (Omega Bio-Tek Inc., Norcross, GA, USA) according to the manufacturer’s recommended procedure. Total RNA quality was detected using agarose gel electrophoresis, and RNA concentration was determined using a NanoDrop 2000 spectrophotometer (Thermo Fisher Scientific, Waltham, MA, USA). The first strand of cDNA was synthesized according to the PrimeScriptTM RT reagent Kit with gDNA Eraser (Takara, Dalian, China) kit instructions, and amplified as a PCR template.

### 2.5. Cloning of NlABCG

Transcriptome data of BPH were downloaded from the NCBI SRA database (SRR8840386-SRR8840388) and assembled using Genomis R9 [33] with reference to the *SfABCG* genes. Using the BLAST tool on the NCBI website for alignment, we confirmed that the assembled genes were *NlABCG*. Gene-specific primers (Table S1) were designed using Primer Premier 6.0 software, and the resulting sequences were verified by RT-PCR under the following amplification conditions: pre-denaturation at 95 °C for 3 min, denaturation at 95 °C for 30 s, annealing at 55 °C for 30 s, extension at 72 °C for 1–3 min for 30 cycles, and final extension at 72 °C for 10 min. The PCR product was tested using electrophoresis and then purified and ligated to the vector for cloning. The cloned products were further expanded and cultured, and sent to a sequencing company (Sangon Biotech, Shanghai, China) for sequencing. The sequencing results were aligned using Blastx.

### 2.6. Sequence Analysis of NlABCG

Nucleotide and amino acid sequence similarity were searched in the BLAST database based on the full-length sequence of *ABCG* genes. DNA MAN software version 6.0 (Lynnon Biosoft, Quebec, Canada) was used to analyze and predict putative amino acid sequences. ORF Finder (http://www.ncbi.nlm.nih.gov/gorf/gorf.html) was used to identify the open reading frames of *NlABCG*, and the “Compute pI/Mw” (http://au.expasy.org/tools/pi_tool.html) in SWISS-PROT (ExPASy Server) was used to calculate molecular weight and theoretical isoelectric points. Pfam (http://pfam.xfam.org/) and SMART (http://smart.embl-heidelberg.de/) were used to identify conserved nucleotide binding and transmembrane domains of all putative *NlABCG* genes. A phylogenetic tree was constructed using the neighbor-joining method in MEGA 6.0 software [34] with 1000 runs.

### 2.7. Expression Analysis of NlABCG under Insecticide Stress

Real-time quantitative PCR (RT-qPCR) primers (Table S1) were designed based on the full-length cDNA sequence *NlABCG*, and cDNA obtained by reverse transcription was used as a template. RT-qPCR was used to detect *NlABCG* expression in each experimental treatment. Reference gene primers were designed based on the cDNA sequence of the BPH 18S gene [35]. Each RT-qPCR was conducted in a 20 μL mixture containing 1 μL sample cDNA, 1 μL of each primer (10 μM), 7 μL diethyl pyrocarbonate-treated H_2_O, and 10 μL FastStart Essential DNA Green Master Mix. The qPCR cycling parameters were as follows: 95 °C for 10 min, followed by 40 cycles of 95 °C for 30 s and 60 °C for 30 s. Melting curve generation was performed from 65 to 95 °C. Three biological replicates and three technical replicates were performed for each treatment.

### 2.8. Data Analysis

The relative expression of 13 *NlABCG* genes after treatment with different insecticides was calculated using the 2^−ΔΔCt^ method. RT-qPCR expression data were analyzed using SPSS 22.0 statistical software, and multiple comparisons were made using one-way ANOVA and LSD methods.

## 3. Results

### 3.1. Toxicity of Insecticides against N. lugens

LC_10_, LC_25_, LC_50_, and LC_90_ to BPH nymphs were calculated as 0.159 mg/L, 0.483 mg/L, 1.653 mg/L, and 17.147 mg/L for thiamethoxam; 0.082 mg/L, 0.181 mg/L, 0.435 mg/L, and 2.308 mg/L for abamectin; and 2.727 mg/L, 4.704 mg/L, 8.633 mg/L, and 27.329 mg/L for cyantraniliprole (Table 1). In addition, the order of toxicity of the three insecticides to the third instar BPH nymphs, based on the LC_50_ value, was abamectin > thiamethoxam > cyantraniliprole.

### 3.2. Identification and Characterization of NlABCG Transporter Genes

**Figure 1:**
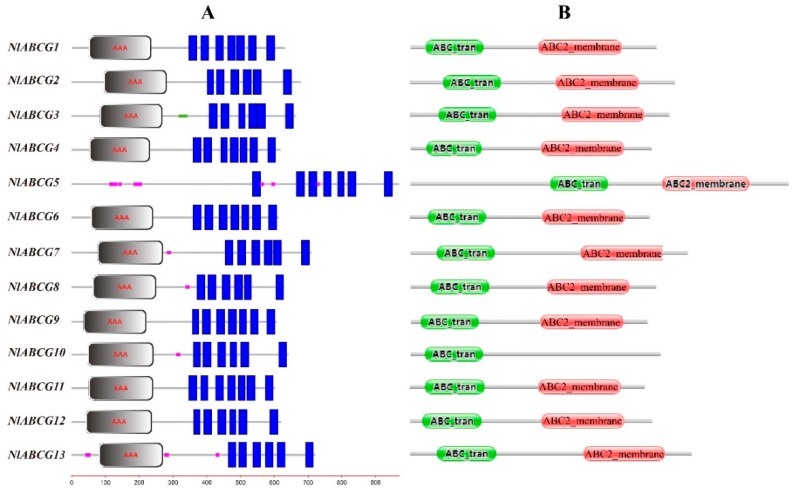
Conserved domain analysis of *Nilaparvata lugens* ABCG transporters. (**A**) Analysis using SMART online software; green marks signify coiled coil; purple marks signify low complexity; blue marks signify transmembrane region. (**B**) Analysis using Pfam online software.

**Figure 2:**
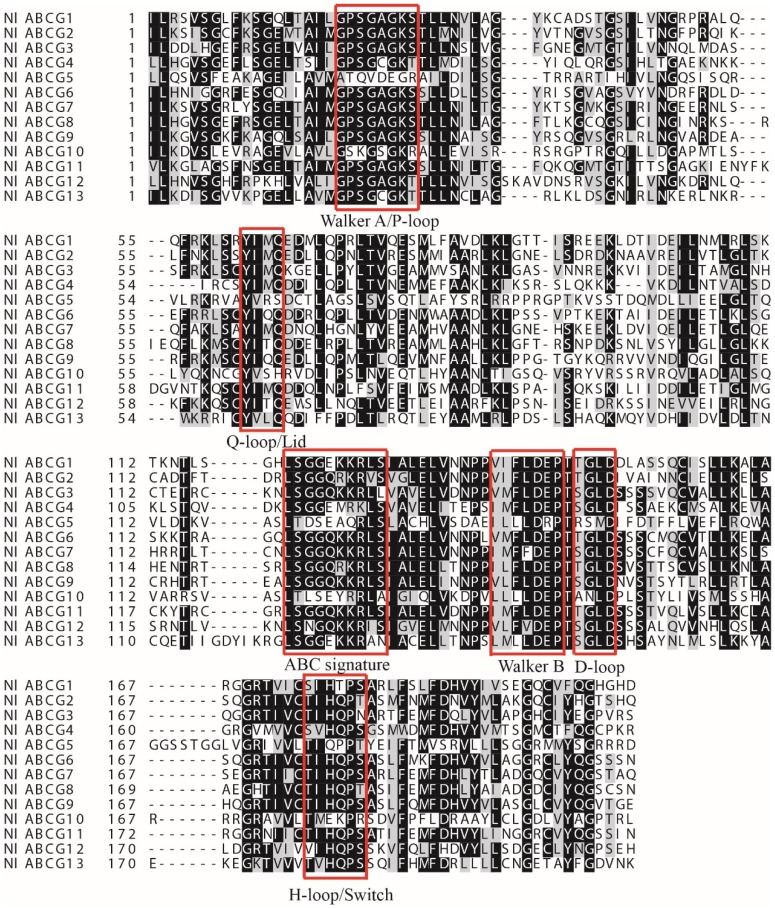
Amino acid sequence alignments of NlABCG catalytic domains. The red-box regions represent different domains.

We obtained 13 *NlABCG* transporters, which we named *NlABCG1* to *NlABCG13* (Table 2). ORFs (open reading frames) of all gene sequences ranged from 603 to 910 amino acids. SMART online software analysis revealed that the *NlABCG* genes contained six or seven transmembrane regions. With the exception of *NlABCG5*, the remaining 12 *NlABCG* genes contained AAA domains (nucleotide binding domains (NBDs); Figure 1A). In order to verify whether there was an error in the *NlABCG5* protein sequence, we analyzed similar sequences from *S. furcifera*, *L. striatellus*, *Bemisia tabaci*, and *Tribolium castaneum*. The results showed that this gene in other species had the same AAA domain deletion (Figure S1) according to SMART software. In contrast, analysis using Pfam online software showed that *NlABCG5* contained an AAA domain and an ABC2 transmembrane domain (TMD), but *NlABCG10* lacked ABC2 TMD (Figure 1B). In addition, sequence analysis indicated that the *NlABCG* transporters genes contained signature C, Walker A/P-loop, Walker B, D-loop, Q-loop/Lid, and H-loop/switch domains (Figure 2).

**Figure 3:**
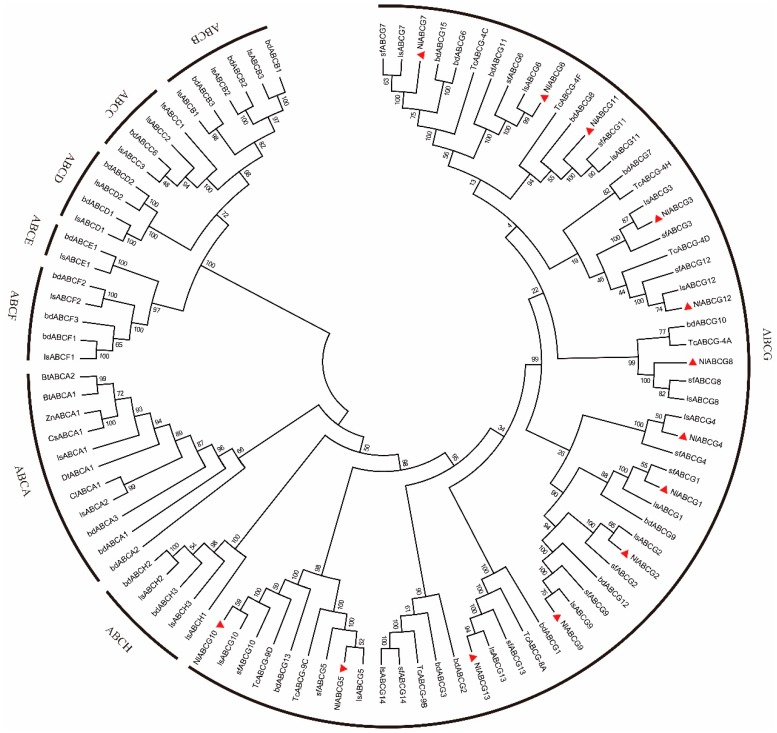
Phylogenetic analysis of ABC transporters in *Nilaparvata lugens* and other insects. Sequences were downloaded from the GenBank protein database. The triangle denotes the ABCG sequence of *N. lugens*.

A phylogenetic tree based on BPH NlABCG transporters aligned with other insect ABC transporter amino acid sequences was divided into eight major branches (A-H) (Table 2, Figure 3). NlABCG from BPH are clustered on the branches of the G subfamily, further demonstrating that the ABC transporters of BPH belong to the G subfamily of proteins. Three species of planthoppers were close to one another, particularly, *L. striatellus* and BPH.

### 3.3. Effect of Thiamethoxam on NlABCG Expression

**Figure 4:**
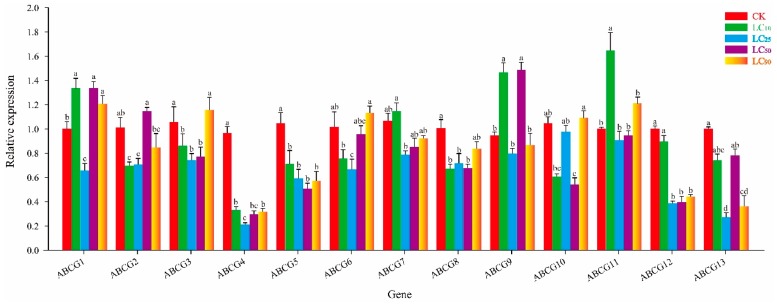
Relative expression levels of 13 putative *NlABCG* transporter genes in *Nilaparvata lugens* under treatment with thiamethoxam LC_10_, LC_25_, LC_50_, and LC_90_. Mean values ± SE were used to analyze relative expression levels under different concentrations of insecticides using the 2^−ΔΔCt^ method; the 18S gene of BPH was used as the internal reference gene, and non-insecticide treatment as a control. Three biological replicates and three technical replicates were performed for each treatment. Different letters indicate significant differences at *P* < 0.05 among different treatment concentrations. LC, Lethal concentration; SE, Standard error.

Under treatment with thiamethoxam LC_10_, expression of *NlABCG1*, *NlABCG9*, and *NlABCG11* were significantly upregulated, while expression of *NlABCG2*, *NlABCG3*, *NlABCG4*, *NlABCG5,* and *NlABCG8* were significantly inhibited (Figure 4). However, expression levels of none of the *NlABCG* genes were significantly upregulated at LC_25_. In addition, *NlABCG1* and *NlABCG9* were significantly upregulated under thiamethoxam LC_50_, and *NlABCG1* was significantly upregulated under LC_90_. With the exception of the inhibition observed at LC_25_, the expression of *NlABCG1* was significantly upregulated under thiamethoxam.

### 3.4. Effect of Abamectin on NlABCG Expression

**Figure 5:**
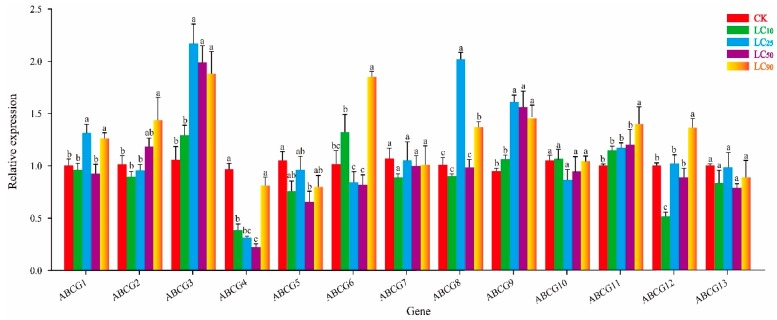
Relative expression levels of 13 putative *NlABCG* transporter genes in *Nilaparvata lugens* under treatment with abamectin LC_10_, LC_25_, LC_50_ and LC_90_. Mean values ± SE were used to analyze relative expression levels under different concentrations of insecticides using the 2^−ΔΔCt^ method; the 18S gene of BPH was used as the internal reference gene, and non-insecticide treatment as a control. Three biological replicates and three technical replicates were performed for each treatment. Different letters indicate significant differences at *P* < 0.05 among different treatment concentrations. LC, Lethal concentration; SE, Standard error.

After abamectin LC_10_ treatment, the expression of only *NlABCG4* and *NlABCG12* was significantly inhibited (*P* < 0.05), while the expression levels of other *NlABCG* family genes changed compared with the control, but the difference was not significant (Figure 5). The expression levels of other genes changed, but not significantly. At LC_25_, *NlABCG1*, *NlABCG3*, *NlABCG8*, and *NlABCG9* were significantly upregulated, while *NlABCG4* was significantly downregulated. The expression of other genes did not change significantly. At LC_50_, *NlABCG3* and *NlABCG9* were significantly upregulated and *NlABCG4* and *NlABCG5* were significantly downregulated. Interestingly, after treatment at LC_90_, eight *NlABCG* genes were significantly upregulated, while the expression of the other five was unchanged. At LC_25_, LC_50_, and LC_90_, *NlABCG3* and *NlABCG9* were significantly upregulated, but these genes were not significantly affected at LC_10_.

### 3.5. Effect of Cyantraniliprole on NlABCG Expression

**Figure 6:**
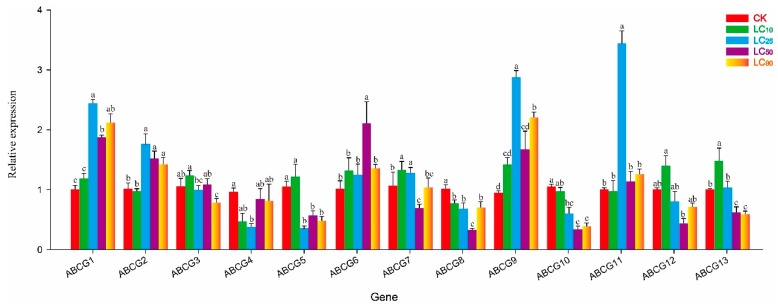
Relative expression of 13 putative *NlABCG* transporter genes in *Nilaparvata lugens* under treatment with cyantraniliprole LC_10_, LC_25_, LC_50_, and LC_90_. Mean values ± SE were used to analyze relative expression levels under different concentrations of insecticides using the 2^−ΔΔCt^ method; the 18S gene of BPH was used as the internal reference gene, and non-insecticide treatment as a control. Three biological replicates and three technical replicates were performed for each treatment. Different letters indicate significant differences at *P* < 0.05 among different treatment concentrations. LC, Lethal concentration; SE, Standard error.

At cyantraniliprole LC_10_, *NlABCG7* and *NlABCG13* were significantly upregulated, while *NlABCG8* was significantly downregulated, and other genes were not significantly affected (Figure 6). At LC_25_, *NlABCG1*, *NlABCG2*, *NlABCG9*, and *NlABCG11* were significantly upregulated. At LC_50_, *NlABCG1*, *NlABCG2*, and *NlABCG6* were significantly upregulated. At LC_90_, *NlABCG1*, *NlABCG2*, and *NlABCG9* were significantly upregulated. At cyantraniliprole LC_25_, LC_50_, and LC_90_, *NlABCG1* and *NlABCG2* genes showed a significant increase in expression.

### 3.6. Co-Induced Expression of NlABCG Genes by Three Insecticide Treatments

**Figure 7:**
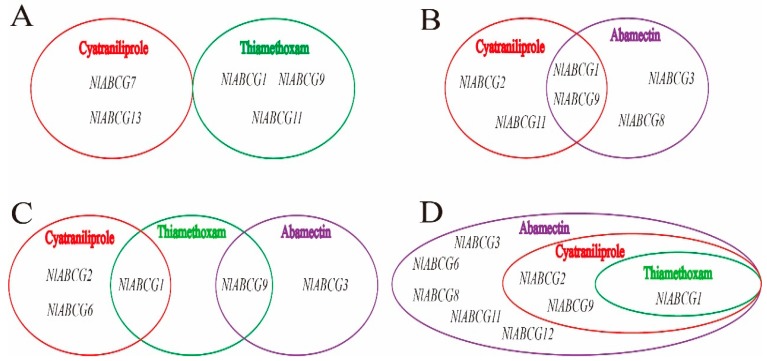
Summary of the significantly upregulated genes in *Nilaparvata lugens* under treatment with thiamethoxam, abamectin, and cyantraniliprole insecticides. The Venn diagram shows the putative *NlABCG* transporter genes found to be significantly upregulated in the insecticide-treated conditions compared with the untreated controls. (**A**) LC_10_ treatment; (**B**) LC_25_ treatment; (**C**) LC_50_ treatment; (**D**) LC_90_ treatment.

To better illustrate the upregulated expression of the *NlABCG* genes under different insecticide treatments, we generated a Venn diagram for the upregulated genes at each concentration (Figure 7). *NlABCG* was not upregulated at LC_10_ of any pesticide (Figure 7A). *NlABCG1* and *NlABCG9* were both significantly upregulated after treatment with cyantraniliprole and abamectin at LC_25_ (Figure 7B). At LC_50_, both cyantraniliprole and thiamethoxam caused upregulation of *NlABCG1*, and thiamethoxam and abamectin caused significant upregulation of *NlABCG9*. However, at LC_50_, no *NlABCG* gene was significantly affected by any of the three insecticides (Figure 7C). Three insecticidal LC_90_ treatments caused significant upregulation of *NlABCG1*; in addition, abamectin and cyantraniliprole also caused significant upregulation of *NlABCG2* and *NlABCG9* (Figure 7D).

## 4. Discussion

ATP-binding cassette (ABC) transporters are an important class of transmembrane transporters. The ABC transporter exists in the form of a full-molecule or a semi-molecular transporter in the organism. The full-molecule transporter contains two NBDs and two TMDs, while the semi-molecular transporter contains one NBD and one TMD [36]. According to sequence similarity of NBD, ABC transporters are divided into eight subfamilies, ABCA to ABCH. Full-molecule transporters are always present in the ABCA, ABCB, and ABCC subfamilies, while semi-molecular transporters are commonly found in the ABCB, ABCD, ABCG, and ABCH subfamilies [20]. In the present study, 13 ABCG transporters were identified and their conserved domains were analyzed and found to have one NBD and one TMD (Figure 1). These transporters constitute semi-transporter proteins, consistent with the findings from *L. striatellus* and *S. furcifera* [19,20]. However, we used Pfam and SMART to analyze the conservative domain of *NlABCG* genes. We found that the results of *NlABCG5* and *NlABCG10* using the two online software were inconsistent. This difference could be attributed to the defects in different prediction software. Therefore, we suggest using at least two online prediction software to analyze gene domains. These studies have shown a conserved sequence of approximately 200 amino acids in the NBD, which contains Walker A (GXXGXGK(S/T), Walker B (XXXLDEP), ABC signature (LSGGQ), D-loop, H-loop, and Q-loop conserved motifs [15]. The 13 *NlABCG* genes obtained in this study had corresponding motifs (Figure 2).

The detoxification metabolic process of insects to insecticides mainly involves detoxifying enzymes and transporters, while ABC transporters are important transporters involved in the transport of insecticide metabolites [37,38]. Additionally, some ABC transporters of members of the G subfamily have been demonstrated to confer resistance to xenobiotics, including insecticides [39]. For example, in *L. striatellus*, numerous *ABCG* genes are significantly upregulated in resistant populations to chlorpyrifos, deltamethrin, and imidacloprid [20]. Meanwhile, LC_10_ and LC_25_ concentrations of thiamethoxam, buprofezin, and abamectin can induce the expression of some *ABCG* genes in *S. furcifera* [19]. Permethrin LD_50_ significantly induces the expression of *ABCG* in *Anopheles gambiae* [40]. Indoxacarb, chlorpyrifos, abamectin, and lambda-cyhalothrin LC_50_ treatment significantly increase the expression of *ABCG* gene in *Plutella xylostella* larvae after 48 h [30]. The results of the present study indicate that the *ABCG* transporter in BPH shows varying responses to different levels of thiamethoxam, abamectin, and cyantraniliprole stress. Similar results have been observed in *Anopheles sinensis* [29], *Bactrocera dorsalis* [22], and *Cnaphalocrocis medinalis* [41]. Although these findings do not directly suggest that the ABCG transporter is involved in the development of insect resistance, our results provide insight into the function of the ABCG transporter. In addition, studies have shown that knocking down the expression of *Drosophila melanogaster* MDr50, Mdr65, and MRP1 by RNAi can significantly increase the sensitivity of *D. melanogaster* to DDT (Dichlorodiphenyltrichloroethane) [42]. Knocking down Mdr65 increased the susceptibility of *D. melanogaster* to nine different insecticides [43]. Therefore, on the basis of these studies and our findings, we believe that the overexpression of the *ABCG* transporter can contribute to insecticide resistance. However, its specific mechanism warrants further study.

Interestingly, after treatment with thiamethoxam LC_25_ and abamectin LC_10_, no *NlABCG* gene was significantly affected, suggesting that *NlABCG* is not involved in the response of BPH to the insecticides at these concentrations. ABC transporters constitute a large family with numerous members. In *L. striatellus*, the *ABCB/C/D* subfamily was involved in the resistance to insecticides [20]. Therefore, in future studies, the genomic data of BPH should be combined to comprehensively study the role of the ABC transporter in its response to insecticide stress.

## 5. Conclusions

In this study, we cloned 13 BPH *ABCG* transporter genes and analyzed their conserved domains and evolutionary relationships. We also determined the expression levels of 13 *ABCG* genes following treatment of third instar nymphs with thiamethoxam, abamectin, and cyantraniliprole at LC_10_, LC_25_, LC_50_, and LC_90_ for 48 h, and identified variable responses in upregulation. Our findings indicate that the BPH *ABCG* transporter is able to respond to insecticide stress and contribute to insecticide resistance in BPH.

## Figures and Tables

**Table 1:** Toxicity of thiamethoxam, abamectin, and cyantraniliprole against *Nilaparvata lugens*.

Insecticide	Toxic Regression Equation	LC_10_ (mg/L) (95% CL ^a^)	LC_25_ (mg/L) (95% CL)	LC_50_ (mg/L) (95% CL)	LC_90_ (mg/L) (95% CL)	Chi-Square Value (*χ*^2^)
Thiamethoxam	Y = −0.275 + 1.261x	0.159 (0.073–0.265)	0.483 (0.296–0.685)	1.653 (1.232–2.220)	17.147 (10.256–37.496)	1.103
Abamectin	Y = 0.639 + 1.769x	0.082 (0.056–0.109)	0.181 (0.141–0.221)	0.435 (0.368–0.513)	2.308 (1.759–3.302)	1.358
Cyantraniliprole	Y = −2.397 + 2.561x	2.727 (0.248–5.158)	4.704 (0.959–7.492)	8.633 (3.988–12.235)	27.329 (18.047–12.974)	5.800

^a^ 95% confidence limit.

**Table 2:** Full-length ATP-binding cassette transporter G Subfamily (ABCG) genes identified from *Nilaparvata lugens*.

Gene Name	Accession Number	Product Size (bp)	Size of ORF (aa)	Molecular Weight	Theoretical pI
*NlABCG1*	MN326305	1917	631	71,025.02	8.66
*NlABCG2*	MN326306	2246	680	75,896.09	9.20
*NlABCG3*	MN326307	2046	665	74,512.47	7.50
*NlABCG4*	MN326308	2004	618	70,498.96	8.91
*NlABCG5*	MN326309	3184	970	106,439.26	9.35
*NlABCG6*	MN326310	2451	615	69,011.27	8.83
*NlABCG7*	MN326311	2263	711	79,592.84	7.14
*NlABCG8*	MN326312	2184	630	71,071.50	8.52
*NlABCG9*	MN326313	1896	607	68,417.12	9.10
*NlABCG10*	MN326314	2343	642	71,090.70	9.12
*NlABCG11*	MN326315	1964	603	68,158.16	8.79
*NlABCG12*	MN326316	1925	621	70,006.10	8.70
*NlABCG13*	MN326317	2298	722	82,264.20	7.86

## Supplementary Materials

The following are available online at https://www.mdpi.com/2075-4450/10/10/334/s1, Figure S1. Conserved domain analysis by SMART online software of Nilaparvata lugens ABCG5. Table S1. The primers of ABC transporter G subfamily gene and *18S* gene for RT-PCR and RT-qPCR. Table S2. Species name, gene name and accession number of Phylogenetic tree constructed.
