# Supplementary material for: Effects of Insecticide Stress on Expression of NlABCG Transporter Gene in the Brown Planthopper, Nilaparvata lugens
Source: Insects. 2019 Oct 8;10(10):334. doi: 10.3390/insects10100334 (PMC6836012; doi:10.3390/insects10100334)
Supplement: Supplementary file 1 [file insects-10-00334-s001.pdf]

Supplementary Material

Effects of insecticide stress on expression of the *NlABCG* transporter gene in the brown planthopper, *Nilaparvata lugens*

Hong Yang [1, 2, †], Cao Zhou [1, †], Xi-bin Yang[1], Gui-yun Long[1], and Dao-chao Jin[1, *]

1Institute of Entomology, Guizhou University; Provincial Key Laboratory for Agricultural Pest Management of Mountainous Regions, Guiyang 550025, People's Republic of China

2College of Tobacco Science of Guizhou University, Guiyang, 550025, People's Republic of China

*Correspondence to: Dao-chao Jin

E-mail address: daochaojin@126.com

Table S1. The primers of ABC transporter G subfamily gene and 18S gene for RT-PCR and RT-qPCR.

Gene	Forward (5′–3′)	Reverse (5′–3′)	Amplification method
NlABCG1	CCAGCAAAATGGATTATCCACC	CGAGCCATCATTGCCGATT	RT-PCR
	AAGTTCAGCGTGGTTACAGT	AGCATCCGTGTATTACCTTCCT	
	GCCAGCCGTTTCAGTTCCTTTG	GCCGCCGTAGATGGACGATATG	RT-qPCR
NlABCG2	CCTTGTTTGATGGGTCCAGAAT	GCACTATCCGAGCCAATAACAT	RT-PCR
	GTCCTGCCACCTACAATCCA	TGATGTTGTGCAACTCTCCAAT	
	TGTCCAGGAGAGGAGCCGTTAG	CATTCTGGTGAGCGTGGTCAGT	RT-qPCR
NlABCG3	ATGTCTGGCATCTTCAAGC	GAATCGGCTGTACTGGTCCAT	RT-PCR
	CTAGTCATAGCCTCATCTGTCC	ACAGTGCCATTACTGACGTA	
	ACACAGGCATCGTTGCTCCATC	CGGCTCGTGAGTTGTCAGGTAC	RT-qPCR
NlABCG4	AGCATTGTTCTCCTATCAGTGT	GATGAGGCAGAATGTTGTGGAA	RT-PCR
	CGGTGCGAATAAGGTTGTTGA	TCACTCTTCATCGACTAGTT	
	GCATGTGGCATAGTGGCTCTCA	GCATCTTGTAGGCGCAGGAAGT	RT-qPCR
NlABCG5	GTGACATCATTAGTGACAGGCT	TTGTGTCTAGCACCTGAGTCA	RT-PCR
	GGCGAGAACCATCCACATAGT	TCCAATTCAACCAATGCTGTCA	
	AGACGGCAAGAACAACGAACCT	TGTCCAGAATGGCACGACCTTC	RT-qPCR
NlABCG6	CAAGCGGATGGTAGTTAGTAGT	AGGAAGCACTCGGTTGATGAA	RT-PCR
	GGTGAGGCATCAATATGGAACT	CTGCCACATAGCCTTCAAGAC	
	AATCTACGCAATGAGTGGTCAG	GCTATTCCAGCCAAGTTCCTTA	
	GGATGAGCCAACTACAGGACTT	AAGGAAGCACTCGGTTGATGAA	RT-qPCR
NlABCG7	TGTGCTTGGTTGTCTGGAACTA	TTGCGGCTGCGATGTCAA	RT-PCR
	TCACGCAGAGATTGGACACT	TGAGCATTAAGGCACTGGATTC	
	GGCTCGGTGAAGGTGAACATCC	TCGGAGACGGAGTAGGTGAGGT	RT-qPCR
NlABCG8	TATCACACTGCGATGCTCTCA	CATTGCGAATCCGAACACTGA	RT-PCR
	GAGCCACAACGCCAGTAAC	AGAGGCAGTCGCTGAACAT	
	TGCTGCCGTTCCTGTCCTCAT	TGGACTCTTGGCGACCATGCT	RT-qPCR
NlABCG9	ACTGACCGCTAGTGTGTAGAG	AGTGAGACAGATGCCGTTGT	RT-PCR
	CTACGAGTGGCTTGGACAATG	ATCAAGTTCTGAGCCGCCA	
	ACGAGTGGCTTGGACAATGTGT	GGCTGGCACTTGGCTGATGTAT	RT-qPCR
NlABCG10	GTGTTTCTGGACAAATGTTCAG	CTTCGTGAGCCTCCTGGTAA	RT-PCR
	CTCAACTGCCTCATCACTTCC	TGCTATACTCATCCACCTCCTT	
	GGCGAAGTGTGGCAAGTCTCA	GGCTCGTCCAATAGCAGCAGAA	RT-qPCR
NlABCG11	AATGACGGAGGAACGTGCTG	ACTTACAACCCATCAGACCAAT	RT-PCR
	ATCGCTACTCAACATCCTAACC	CCATTCAACAGGTCCATTCTCA	

	CTCAAGCACGGTTCAACTGGTT	TCGTCCGCCATTGATGAGGTAA	RT-qPCR
	CCTACTATGCTGTAGTGACTAC	CGACGGCACAATGAAGATG	RT-PCR
NlABCG12	TCCTTCTATTTCTTCGCCGTAT	AACCACTAGAGTGCAGTTGAAG	
	GGCTGGTGCTCTGCTCAATGTC	GCTGCCGCAACTAAGCGTCTT	RT-qPCR
	ACCATCACTTGCCTCACAGACC	CATGACAGCCAACAGCTCTCCT	RT-PCR
NlABCG13	GCGATGATGACTCGTGACAT	TGAGCGAACAACATCCAATACG	
	TCATCACAGCGGCAAGAGAA	TGTTGGCGAGTTCACTTGG	RT-qPCR
NL18S	GTAACCCGCTGAACCTCCT	TCCGAAGACCTCACTAAATC	RT-qPCR

Table S2. Species name, gene name and accession number of Phylogenetic tree constructed.

Species name	Gene name	Accession number
Bactrocera dorsalis	bdABCG15	ATY74535
	bdABCG2	ATY74528
	bdABCG1	XP_011207703
	bdABCG12	ATY74524
	bdABCG11	XP_011206695
	bdABCG10	XP_029407401
	bdABCG9	ATY74521
	bdABCG8	XP_011214717
	bdABCG7	ATY74519
	bdABCG3	XP_011203680
	bdABCG6	XP_011210029
	bdABCA1	XP_011203262
	bdABCA2	ATY74533
	bdABCA3	ATY74505
	bdABCB1	XP_011209560
	bdABCB2	ATY74509
	bdABCB3	ATY74510
	bdABCC6	ATY74531
	bdABCD1	XP_011200700
	bdABCD2	XP_011208517
	bdABCE1	XP_011201626
	bdABCF1	ATY74516
	bdABCF2	XP_011212094
	bdABCF3	XP_011208050
	bdABCH2	ATY74526
	bdABCH3	ATY74536
Tribolium castaneum	TcABCG4A	XP_008192053
	TcABCG4C	XP_001813184
	TcABCG4D	XP_008192597
	TcABCG4F	XP_971735

	TcABCG4H	XP_973526
	TcABCG8A	XP_975214
	TcABCG9B	NP_001034521
	TcABCG9C	XP_968472
	TcABCG9D	XP_968555
Sogatella furcifera	SfABCG1	QAV55724
	SfABCG2	QAV55725
	SfABCG3	QAV55726
	SfABCG4	QAV55727
	SfABCG5	QAV55728
	SfABCG6	QAV55729
	SfABCG7	QAV55730
	SfABCG8	QAV55731
	SfABCG9	QAV55732
	SfABCG10	QAV55733
	SfABCG11	QAV55734
	SfABCG12	QAV55735
	SfABCG13	QAV55736
	SfABCG14	QAV55737
Laodelphax striatellus	LsABCG1	AIN44110
	LsABCG2	AIN44111
	LsABCG3	AIN44112
	LsABCG4	AIN44113
	LsABCG5	AIO05329
	LsABCG6	KF828797
	LsABCG7	AIN44115
	LsABCG8	AIN44116
	LsABCG9	AIN44117
	LsABCG10	AIO05330
	LsABCG11	AIN44118
	LsABCG12	AIN44119
	LsABCG13	AIN44120
	LsABCG14	AIN44121
	LsABCH1	AIN44122
	LsABCH2	AIN44123
	LsABCH3	AIN44124
	LsABCA1	AIN44094
	LsABCA2	AIN44098
	LsABCB1	AIN44099
	LsABCB2	AIN44095
	LsABCB3	AIN44096
	LsABCC1	AIN44102
	LsABCC2	AIN44103

	LsABCC3	AIN44104
	LsABCD1	AIN44106
	LsABCD2	AIN44107
	LsABCE1	AIN44108
	LsABCF1	AIO05328
	LsABCF2	AIN44109
Cryptotermes secundus	CsABCA1	XP_023727470
Zootermopsis nevadensis	ZnABCA1	XP_021941981
Bemisia tabaci	BtABCA1	XP_018909306
	BtABCA2	XP_018898232
Dinothrombium tinctorium	DtABCA1	RWS10623
Cimex lectularius	ClABCA1	XP_014253338

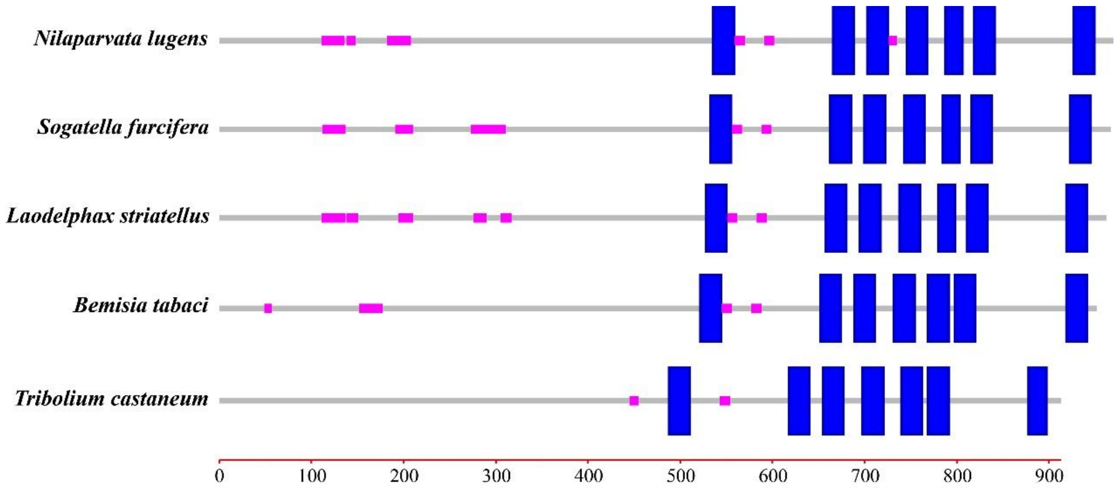

Figure S1. Conserved domain analysis by SMART online software of Nilaparvata lugens ABCG5.

Gene Sequence

>NlABCG1

ACCAGCAAAATGGATTATCCACCCGCAGTGGCCAACATAAACCATCTAATCCAGAGG
CCACCGGTTGATATAGAGTTCACAGATCTCACCTACACAGTTCCTCATGGGAGAAGTG
GTTCAAAGATTATCCTTAGAAGTGTGAGTGGTTTATTCAAGTCGGGACAATTGACTGC
TATCCTGGGACCCTCAGGAGCCGGGAAAAGCACCCTTCTAAATGTACTAGCTGGATA
TAAGTGTGCAGATTCTACCGGCTCCATCCTAGTCAATGGCCGCCCTCGAGCTCTGCAA
CAGTTTCGTAAGTTGTCTCGCTACATAATGCAGGAAGACATGCTTCAGCCCCGCCTAA
CAGTGCAGGAATCCATGCTGTTTGCTGTCGACTTGAAGCTTGGAACCACCATTTCACG
GGAGGAAAAGCTGGATACTATTGACGAGATTCTGAACATGTTGAGGCTTTCAAAAAC
GAAGAACACTTTGTCTGGTCACTTGTCTGGTGGAGAGAAGAAGAGACTGTCCATTGC
TCTCGAACTGGTGAACAACCCACCTGTTATCTTCCTTGATGAACCAACAACGGGCCTA
GACGACCTGGCCAGCTCTCAGTGCATATCCCTTCTGAAGGCATTGGCGAGGGGCGGT
CGCACAGTCATCTGCTCCATTCACACGCCGAGCGCCCGCCTCTTCTCACTGTTCGACC
ATGTCTACATCGTGTCGGAGGGCCAGTGCGTCTTCCAGGGCCATGGCCACGACATTGT
CGCCTTCCTCGCCTCGTTCGGCCTCAACTGTCCCAAGCACTACAATCCCGCCGATTTC
ATGGTTGAAGTATCTAGTGGAGAGTATGGTGATTATCTGGAGAGAATGACTAATGCC
ATTGAGAATGGGAGGTGCTATAAGTGGAATCAGAACAAAGTCACTGACGTCCGATAC
CAAGCAAATGAAGAGGAAGAGAATCTAGTCAGTACTGATCTCCATCACATGTACAAT
TTCGAAAGTTCAGCGTGGTTACAGTTTAGGATATTAATAAGTAGAATGTCTCTACAAG
GTAGAAGAGATATGGGATATATTATATTGAAATTAGCAATGCATATTTTCATAGGAAT
GATTATTGGAGGGATGTTTTTCCAAATCGGCAATGATGGCTCGAAGACTATTTTCAAT
TTTGGTTTCTGTTTTGTTACGATTATCATTTTCTTGTATATACCAATGATGCCAGCGCTT
CTATGGTTTCCTCAAGAAGTGCAGTTATTGAAAAGAGAATTTTTCAATAGATGGTATG
ATCTGAACCCGTATTTTTTCGCCATGACATTCTGTCAGTTGCCGTTGCAAATTGTTTTT
GGAATCGGGTATGCACTGTTAACGTACTTCATGACAGATCAGCCAATGGAGTATGAG
AGAGTTCTCAAATTCATTCTGGTGTGCCTGATGATCTCCATTGTGTCAGAAGCCATGG
GGTTGGCAATATCTGCTCGTCTCAATATTGTCAACGGCATTTTCGTGGGGCCAGCCGT
TTCAGTTCCTTTGATGTTACTGGCGGTGTACGGCCTAGGCACGGGGAGCAAATACATC
CCTTCGCACATCCGTTTCGCCATGTACTTCAGCTACCTGCGCTACGGACTGGAAGGCC
TCATATCGTCCATCTACGGCGGCGGCCGCACCAAAATGGTCTGTCCCGACTCCGAGAT
CTACTGCCAACTGCGCGAACCTAAAGCGCTTCTCAAGGAGGTTGGCATGGAAGACGT
CAACTATTGGTTAGACATTGCTGCGTTGGCAGTTTCTTTCCTGGTCTTCAAGGTCATAT
GCTATGTGCTCTTGAGGAGGCGTTTGAAGTCCACGCAATCGTTTGGCGCACTCGGGTT
TATCGGCAGATTTATCAAGACCCATTTCAATCTAGCTGGAAATATAGGAAGGTAATA
CACGGATGCT

>NlABCG2

CCTTGTTTGATGGGTCCAGAATTATTATTGTTGAAAAATTGGGAGATTGAAATCCATGA

CATTTAAATTTATTCTGAACTAAATATAGTAGTTCTGAAGCTGCACATATCTTCTGCATTA
GACTTGCCAATAGTTTGATATTTAGAAAAGTGTTAAAATGACAGCGTCCACATCATCAG
CATTGGAGAATATTGTTGAAGTTTTGCCATGTCCAGGAGAGGAGCCGTTAGCGGGGTA
CAGCGGCGAAAGGGTCACCAAGGGGGCACTAGCGACTGCCAGCCGTCATCCACCCGT
ACCCGTCACAAGGATTCCAACCCGGCTCGGACTGACCACGCTCACCAGAATGGCCAA
GCGACCCGCTGTCGACATCGAGTTCCAGGACCTCTCTTACACAGCTGGAAGTCGGAAG
ATTTTGAAATCGATATCAGGATGCTTCAAGTCAGGAGAGATGACCGCTATCATGGGTCC
TTCGGGAGCTGGAAAGAGTACCTTGATGAATATCCTTGTCGGTTATGTAACAAATGGAG
TGTCGGGAAGTATTCTGACGAACGGATTTCCACGACAAATCAAGCTGTTTAATAAGCTG
TCTTCGTACATAATGCAAGAGGATCTACTACAGCCAAACCTCACAGTCAGGGAATCCAT
GATGATCGCAGCTAGACTCAAACTCGGAAACGAATTGTCTGACAGAGATAAGAATGCT
GCAGTGCGAGAAATCCTTGTGACGTTGGGCTTGACTAAGTGTGCGGATACATTCACAG
ACAGACTGTCGGGTGGCCAGAGAAAGAGGGTGTCCGTGGGACTCGAACTCGTCAATA
ACCCGCCAGTTATATTTTTAGATGAACCAACCACGGGTCTAGACATAGTAGCCATCAAT
AACTGCATAGAACTGTTGAAGGAGCTGTCAAGCCAGGGCAGAACAATCGTTTGCACCA
TTCACCAGCCGACTGCTTCCATGTTCAACATGTTCGACAATGTGTACATGCTTGCCAAG
GGCCAATGCATCTACCATGGCACTTCACACCAACTGGTGCCTTTTCTTAGTAATTGCAA
CCTCGACTGTCCTGCCACCTACAATCCAACTGATTTCGTTTTCGAAGTTTTGGAATCAA
ATGCAGAATTTATCAAAGTAATGAACTCTGAGATACAAAATGGTAGAGTCATCTGGTTG
GATCCAACCGATAATCCTGAGCCAAAGTCAAAATTGTGTAGAAAAGATACACTAGCTG
TTATACCCAATGTTATTGGCTCGGATAGTGCAATACACTTCCCGACAACATTTTTCGAAC
AAGTAACGATATTGCTGAGAAGAATGATGAAACAGAAATGGAGGAATTCGACTGCCAT
GAGGTTGCAGATGATCCATCATTTGTTTTCTGGACTGATTGTTGGTTCCATATTCTATGG
AATTGGAAATAACGCCAGCAAGCCGTTTGAAAACTTCAAATTCGTTCTTTGTGTTGCTG
TTTTCTTCATGTACACACATGTTATAACACATATCTTGACTTTGCCAAATGAAATCAAAA
TCATGAAAAGAGAGTATTTCAACCGATGGTATGGACTCAAAGCCTATTTCACGGCGCTC
ACCCTTCATACAGTTCCTACTACCATAATTTTGGGTATGATATTCAACACAATCGTATACA
TAATGGCGGATGAACCATTAGAGCTGCCCAGGTTTATATGGTTCAGTTCATTCACAATCA
TGGTGGCTCTTGTCTCAGAAGGACTCGGAGTGCTCATCGGATGTAACTTCAACTGCAC
TAACGGAGCCGTAGTGGGTCCATCAGTAATGGCACCAATACTTATGATCGCCATTCACG
GAATGGGGTACGGATTACACATAAAGCCGTTCATGAAGAGTCTGATGAACATGAGCTT
CATTCGCATAGCAGTGGTGGGCATAGTGACGAGTCTCTATCAGAATGGGCGTGGCCCGA
TGGAGTGCAAAGCCCAGGTGCATCCCTACTGCCACTACCGCGACCCCTACATGCTGGT
CAGGGATCTCGGCATGACCAATCAGAGCTCGGTCAATCAGATTATCGGCCTAGTCGGCT
TTCTACTCTTGTTCAGGATGGCCGCCTTTTTGACTCTCAGGTACACACTCATGACTGAC
ATTAGAAGTCAAGTGTTCGCCTATACTAAGAAAATATTCAAACGCAACAAGGAGAAGC
GTATGTTGTTGTCTGATGAGAAGTAGAAAAATCAATCGTTATCAAAAAATATTGGAGAG
TTGCACAACATCA

>NlABCG3

ATGTCTGGCATCTTCAAGCTATTCAAACTCCAGAATGGGAGCGTCGAAAAAGATATGTC
ACATAATATCTACACAGTGGACCTGACCCAGAGTGACACAGGCATCGTTGCTCCATCCT
TCTCGCTCAAACATCTTCCGAAGAGACAGCCTGTTGACATCAAATTCTCTGACTTGAAT
TACACTGTACCTGACAACTCACGAGCCGGTTCGAAAAAAATTCTGGATGATTTACACG
GAGAATTCAGGTCTGGAGAGCTGGTGGCAATATTGGGGCCATCAGGCGCTGGCAAAA
GTACTCTCTTAAACTCCCTAGTCGGTTTTGGAAATGAAGGAATGACGGGAACAATATTG
GTGAACAATCAGCTGATGGATGCCAGTTCGTTCCGCAAGCTAAGCTGCTACATCATGCA
GAAGGGCGAGCTTCTCCCTTATCTGACAGTCGGTGAGGCCATGATGGTTTCAGCCAAT
CTTAAACTGGGAGCTTCAGTCAACAACAGGGAGAAAAAAGTTATTATAGATGAGATTC
TTACAGCTATGGGACTTAACCACTGTACGGAAACTCGATGCAAGAATCTGTCGGGTGG
TCAGAAAAAGCGATTGCTTGTAGCTGTGGAGCTTGTAGACAATCCACCTGTCATGTTCC
TGGATGAACCAACAAGTGGCCTGGACAGCTCGTCGAGTGTGCAGTGCGTCGCGCTGC
TGAAATTGCTGGCGCAAGGAGGCCGAACAATAGTGTGCACCATACATCAGCCGAACGC
GCGCACATTCGAGATGTTCGACCAGTTGTACGTGCTTGCACCGGGCCACTGCATCTATG
AGGGCCCAGTACGCTCGTTGGTGCCCTTCCTCGCTAGTCATAGCCTCATCTGTCCCAGC
TACCATAATCCGGCAGATTTCGTTATTGAAGTTGCTATGGGACAACATGGACCAGTACA
GCCGATTCTTACCAAAGAGATAAAAAGGCACATGAAGGAAAAATTCAAGGAGGAGGA
GAACAGAATATCTGACAAGATCAACGAAATTGATCAGCCAAACAATAAAAATTCTATTT
CAGGTGAAAGATTTAGAGTATTATCGAGTGAGCTTGAGCTATCTCCAAAAGATGAGAAT
TCGTTCAATCGCCTTAGGGGCGATGGGGAGTTTGATGTTTGTGAGAGGCCATCAGCTTT
ATTTCAATTCTATATTCTATTGAAAAGGACATTCACCTCAACTTCCAGAGATCTTCAACT
GACCCAACTGAGATTGGTATCGCATTTTGCGATCGGACTTCTGATTGGCTACCTCTACCT
CAACAAAGGCCAGGACGCCAGCAACATCACCAACAATGCTGGCTGCATATTCTTCACA
GCCATGTTTCTTATGTTTTCATCCATGATGCCCACTATTCTAACATTTCCATTGGAGATGA
CAGTCTATAAAAGAGAGCATCTGAACAATTGGTACTCACTTGGCCCATTATATTTGGCG
AAAACGTTGGCTGACATTCCATTCCAGGTGGTGTTCACAGTAGTCTATGTGTCAATAGT
CTACTATATGACCGATCAGCCGCAGGATTTGGAACGGTTCTCAATGTTCTTATTCGTGAG
CATTTTACTGTCTTTGGTGGCATCATGTGTTGGCCTTTTGACTGGGACAGCTCTATCAAT
AGAAACAGGCACCTACTTCGGACCAATCTCATGCATTCCCTGCGTCCTGTTCTCCGGCT
TTTTTCTGTCACTAGACTCAATTCCGTCCACAATGAAGTGGCTGGGCAGCCTCTCCTAT
CTGCGCTACGCGTTCGAAGGCTGTATGCTCTCGCTCTACGGCTTCGACAGATCCAAACT
CGACTGCTCTGAGGTCTACTGCCACTTCAGAATGCCATCTCAGTTCCTCAAACACCTCG
GCATGCGGGATGCCTCCTATTGGTACGACTGTAACATTCTAATCATTTTCGTTGTGGTGC
TCAGAACCTTGACCTACTTTGTGCTCAAGTATAAAATCAAGTCTTCCTCGTCATAGACA
TCGAAATTCCAGTCGATAACACTTTTACGTCAGTAATGGCACTGT

>NlABCG4

AGCATTGTTCTCCTATCAGTGTTTGAAACTTTAAATTTTTCGCTATCGTCAGTCGCGTA
CATTAAACCAAAGAAAAGAGAGTCTATTGAGTCGTTGTAAATTACTTTGTGATTATAT
ACGAATCACACATGCCGACAGTTTATCAGAATGACTTCAGTGATAGATTGTTGTTCCT
CTCCGAAAGACAATAATTTATGTTTAACGTTCAGTGAGCTGTCGCATGAAGTCAGATG
TGGTGAGGGGTTTCGATTCATGAGAGACAGAAAACGCTTACTCCATGGAGTTTCTGG
GGAATTCCTATCCGGAGAGCTTACAAGCATTATTGGCCCATCAGGATGTGGAAAAAC
CACGCTCATGGATATCCTATCTGGTTACATACAATTGCAGAGAGGATCGATTCATTTG
ACTGGAGCTGAGAAAAATAAAAAGATTAGATGCAGTTACATAATGCAAGACGATATT
CTGCAGCCTCTACTAACTGTAAACGAGATGATGGAGTTTGCTGCCAAACTAAAGATT
AAGTCAAGGAGTCTTCAGAAGAAGAAAGTCAAAGATATTTTGAATACAGTTGCGCTC
TCAGACAAATTGAGTACACAAGTCGATAAACTGTCTGGAGGTGAAATGAGAAAACTG
TCAGTGGCTGTCGAGCTTATCACTGAGCCATCAATCATGTTCCTAGATGAACCTACCA
GTGGATTGGACATATCGTCGGCAGAGAAATGCATGTCGGCATTGAAGGAAGTGGCTG
GCAGAGGGGTGATGGTGGTCTGCAGCGTGCACCAGCCCAGTGGCTCCATGTGGGACA
TGTTTGATCACGTCTATGTAATGACCAGTGGAATGTGCACGTTTCAGGGTTGTCCCAA
GAGATTGATGGATTACCTGCACTCACTAGCTCTTACCTGTCCCATGAATTATAGCCCG
GCTGACCACATTCTAGAAATTACTCTTGAAGAATATGGAAATAATCTTTCACGATTGG
TGAGTGCATCTAGAAATGGAGCAAATAAAGAATGGAGACGAAGCAACAGACAAATC
CCTGATGAAGGTGATATCTCGCTTTGTGATTCAGAAAAGTTAAAAGTTTCATCCTATG
GAGTGGAAAGGTTTCCAATATCGTCCATAATGAGACAAAAATGCTATTCGCCTCCATT
TATAGTTCAATGCATTGTTTTATTGGAGAGAGGTGCAAAAAGTCTGTGTAATTCAAAT
AATGAACCGGTGCGAATAAGGTTGTTGATCCATGTGTTGATGGGATTATTCTTTGGAA
GCATCTACTGCAGAATAGGCCTGGATGCAGCTCATGTACGCGACAACCATAGCCTTCT
TTTTTACACTTTGATTTTCATCATGTTCACTGCATACTCAGGAATGATTATATCCTTCC
ATCTTAAACTTCGGATAACCACTCGTGAATATTTTAATAAGTGGTACTCATTGAAAGC
ATTCTACTTGGCTGAGAATATTATGGATATGCTCTTCCAGATATTATGTTCCACAACAT
TCTGCCTCATCATTTACTTGTTATCAGGACAACCTCTGGACCCGTTGAGGTTTTTCTG
TTTGCCTCAGCATGTGGCATAGTGGCTCTCATTTCTCAAACGTTGGGTCTATTGGTTTG
CACAATCTTAAGACTGAAGCATGCAGTAGTGTTCGGATCATTGTTTATCATGCCTTGG
GTGATATTTTCGGGCTACTTCCTGCGCCTACAAGATGCGCCGTGGTTCTCGCACTGGC
TTTTCCACATCAACTTTCTCAAATACGGTTTCCAGTGTGTGGTTCTGTCGATCTACGGC
TACGATCGACCGCGCATGCCCTGCTCCAAAGACTATTGCCACTTTGTGTTTCCACAAA
AGTTTCTCAAGCACCTTCAACTGCAGCAAGAAAAGTACTATGCCAACTTTTTGATTTT
GGCGGCCATTCTTCTAGTTACCAAGATTCTCACATTCCATGTGCTCAAATACCAGCTT
AAACATAAACGAAAAAAACTAGTCGATGAAGAGTGA

>NlABCG5

GTGACATCATTAGTGACAGGCTATGAGAATTCTCTTAAATCCATTACATAGTAAATCC
TTCTGTTATCTATAGTTGAAGTTCATTAGTCTCATAGTATCAATTCAAATCCATTGTAC

GTTCATCGGAGCCCTTCTGAATAAATAAAAAGACACAAAATCAGACCGGATGCAAAA
CTGAATAATGGCGGACGCAGTAAACTTATGACGTTGTGACAGTCCTGCAAAGGAAAT
TCAAGATGGTGGGTCGCCAACAGAGGGATATGGAGAGGAGGTACTCCATAGCAGAA
GTTCCTTCGGAGCTGAGTGGAATGCCTCCACCAGGGTTGATGCCTTCAGCCTCTGAGG
ATCTCCATGCTTGGTCTATCTACAGGCAAAATCTGAACTCAGATTTCACAGACTCGGC
CCTGGGCTCCAGTGAGAAGTCGCCTCTACCTTACGGCAACTTCCAACTCAGGGAATCA
ACGGTGCAATCTATCCTCAGTCATCCCCGCTATGGACCCAAATCAGCGCTCGGTTCCA
ACATGTACACGTACCTGAAGTTCGGCCTACCTCGGGTGTTTCCGCCGAATGGTGTGAG
GGGCGGAAGAGACGGCAGCAGCGGCTACGACTCCAGTGATGATGGTGGCGGTGGAG
GGGGGAACGGTACTGCAGCCAGACCGCGACCCAGAGCTAGACATGCAAGTCAGCAA
CATTTGCACGCCCCACCCGGATACTATTTACGCGCCAGGAGTGATCCTGATTTTCGGA
ACACGCCGTATCATGGGCCAAGCATGCCCCTGAGACAACAGATGGGTGGCCCCGGGG
GCGGGGGTGGAATGCCGCCTCCCCCCCACCAGCAGCACCCAGCCGGGGGCCGTGGCA
AGAGTGTGAGTGAGGCCAACCTGCTCGCACCAGAACTGCTGATGAGGCATAATGCAG
CTCCATATGAGCACAGGCGCAGTGTACATGATCTGCGGGGCGCAATCGCTTACTCAG
AGCTCGGGGGACCACCGCCGCATGTATTGGTGCATAATGCTAGACACGGAGGTCGTC
CAGCTTCCGTGGCAGTGGTGGGAACAGGACACCACCACCCGGCAGTGTTACACCGCC
ACGGCAGTCACTCGGTGTTGGACGGCGGAGTGGGGGGTGGTGCCGCCCCCAGCATGC
TCAGTGGCATGGCACCCCCCTCCTCACGAGCCCACTCAGCCCTCCGCTCTCATGACGC
ACCGCCAGGCATGTCCTTCCAGGTGCATCGTGGGGAGGCGTTTAGTGGAGCCTATCC
GCATTTGCAGGTTCGAGGTCTAGATGTAGACGGCAAGAACAACGAACCTCTGCTCCA
GTCGGTTTCGTTCGAAGCCAAAGCTGGAGAAATCTTAGCAGTCATGGCAACACAAGT
TGACGAAGGTCGTGCCATTCTGGACATCCTGTCGGGTACAAGACGGGCGAGAACCAT
CCACATAGTGCTGAACGGACAGAGCATCAGCCAGCGAGTGCTGAGGAAGAGGGTGG
CCTATGTGAGGAGTGACTGCACCCTGGCGGGGAGTCTCAGTGTGTCGCAGACGCTTG
CATTCTATTCAAGACTCAGGAGGCCTCCCAGGGGGCCCACCAAAGTATCTTCCACCG
ATCAGATGGATCTGCTAATAGAGGAGTTGGGCCTGACTCAGGTGCTAGACACAAAAG
TGGCAAGCCTGACCGACTCGGAAGCGCAAAGACTGAGCTTGGCCTGCCATCTGGTGT
CCGACGCCGAAATTCTGCTTCTGGATCGCCCCACGCGGTCCATGGACATTTTCGACAC
TTTCTTCCTCGTCGAGTTTCTACGACAGTGGGCTGGAGGTAGCAGTACAGGTGGTCTA
GTAGGCAGAATAGTGGTGCTGACCATCCAGCCTCCAACCTACGAGATCTTCACGATG
GTGTCGCGGGTGCTGCTGCTCTCTGGTGGCAGAATGATGTACTCGGGCCGAAGACGA
GACATGTTGCCATATTTCTCCGCCGCTGATTATCCCTGTCCTGCCTTCAAAAATCCTTC
CGATTATTACCTTGACCTGGTAACCCTGGATGACCTTTCGGCAGAGGCGATGCTGGAG
TCGTCGCAACGCATCGAGCAGTTGGCAGAACTGTTTCGGCGCCGACAAGAGCCCCTC
TCCGACCCGGGACCACCGCAGGCCCTGCCTGGCAAGACCAGGACTGCCAACCTATGC
TCACAGGCTGTTGCTTTGCTCATGAGACAATTGATCTACTCACAGCCGACCAGCCTGA
CCAACTGGTTGACTCATGTTCTTCTCGCTGCCATACTTTCACTTATTGTTGGTGCTATA
TTTTGGGATGTGCCCAAGTCTGATCCGCAACTTCTCTATGCTGACAGGATTGGATTCC

ATTATACAATGATGTGCGTTGCTTCACTGCCCATTCTACTGATGCTAACTCTGAGCGA
CGCTCGCAGTTCTGAGAGAGCAGCTTCCGAGATGGATATCAGAGATGGTCTCTATTCG
AGGCTCATTTTTATCATTATAACAGCTATTATCAGCTTTCCAGCCGTGCTGTTTGTTTG
GCTTGCTTATATAATTCCAGCCTACGCGATGACTGCATTATATAATCAGGGCTCACAA
ACACCCAACGGATTCCACATCTATATAAGCACAATGCTGGTACACATGATGTGCCTCT
ACTACACACTGAGGCTCATCAGCCAACTGTGGCGGTCCCGGCGGACGGCGGCCATCG
CTTCCGGTCTGGTGTTGGTGGTGTTCTCGCTGGTGTCCGGATATCCGGTGTACCTGGC
GGATGTGCCCCCCTGGCAGGCCAACTACTTCGGCCTGGTGTCTCCCGTGAGATGGATG
ATGCCCAGTCTCCTAGCCAGAGAGTACTCCCCGGTCACTCTGGCCGCCATTGCTTCAC
AGATGATTTGCAACAATCGACAGGTCCAGCAGCAAGACATCATAGTCCAACTGCCCT
GTCCTATACCGAACGGCACTGCTGCTCTCTCTTTCTACGGTCTCTCTCCCAAGTCAGCT
GTACCCTTCAACTGGACGCAAGTACTGCCCTACTGGCCGCCAGTGATTATTGCCCTGG
CTATGGCTGTTCTGCATACTGTCATCTTCCTCTTCAGATCCCCGACTCCGGCGTGGAA
GAAAGAAGATAAACTGAAAAGATATATCTATCATCCTCACTAGATTCACATCAAACT
GACAGCATTGGTTGAATTGGA

>NlABCG6
CAAGCGGATGGTAGTTAGTAGTCTAGTCTGTAGACTCGTGCGCGCATGTCTCTCGTCA
ACAGATACTTCCATAAATCAGTTTAAATTTTTTCTCCAATTTATGACTGCCACGTTTCA
CCAACTATCCACTAACAATTTGTTGATCTCTGCGTTTATTGTGAAGCGCGTGTGCATTC
GCGAAAACAAAACACACAACAATAATTCAGAACATTACAAATTGACTCCATTGTTCT
TTTTATTTGTTAACTTCAGTTAGTGTGTTTTTGTTATTGTTTACCTGAGCTTCTGTTCTT
ATTTCATCGCTCGCTCGCCAAGTTTCAAGGTGAGGCATCAATATGGAACTGGAAACA
GAGCTGAGGTCAACCTCAGCGGGAACCTATTTCTCGAGGCATGCAGCTAGGGAGCCA
GTCGAACTCAGCTTCGACAATCTCACCTACTCTGTTTCACAGGGTTTCCAAAAAGGCT
CAAAAACTATACTACACAACATTGGAGGCAGATTTGAATCAGGTCAAATAATTGCTA
TTATGGGACCTTCAGGAGCAGGAAAATCATCCCTACTGGATCTCCTATCAGGATACA
GGATATCTGGTGTGGCGGGATCTGTCTACGTGAATGATCGATTCCGAGATCTGGATGA
ATTTCGAAGACTGTCATGTTATATACAGCAGGACGATCGACTGCAACCTTTGCTGACT
GTTGATGAGAACATGTGGGCAGCTGCTGATCTCAAATTGCCTTCAAGTGTCCCCACAA
AAGAGAAAACAGCTATTATAGACGAGATTTTAGAGACCTTGAAACTCTCAGGCTCTA
AAAAGACCAGAGCCGGACAACTTTCTGGAGGACAGAAAAAAAGACTTTCAATCGCAT
TAGAACTAGTGAACAATCCATTGGTAATGTTTCTGGATGAGCCAACTACAGGACTTG
ATAGCTCTTCGTGTATGCAGTGCGTCACATTACTGAAAGAGCTCGCATCTCAAGGTAG
AACAATAGTCTGTACAATTCATCAACCGAGTGCTTCCTTGTTCATGAAATTCGACCAC
GTTTATGTCTTGGCGGGTGGAAGGTGCCTTTATCAGGGATCTTCAAGCAATCTTGTTC
CATACCTGGCCGATCTATCGCTACCGTGCCCGACCTACCATAATCCTGCTGATTACAT
TATTGAATTGGCCTGTGGAGAGCATGGAGAAGACAAAATTGATAAACTAGTGGATGG
AACACAGAACGGCAAATGCTATAAATGGTTCACGAATGGAGAAGTATTGAAATACAA

CAATAATGCTGCAGCTGATGTCACTTCTATGTCATGTCTACCCATTATGAAAAAATGT
GGAGGCTCACTTCAAGTCACATCTCAATGGAATCAAATCAGTGTCCTTTTGAGAAGA
GGATTCATCAAAATGAAAAGAGATCAGACTCTAACTCACATGCGGTTCATGGTGAAC
GTACTGACTGGGATGATGTTGGGAGCGCTGTTTTTTCAAACTGGCAATAAAGGGGAG
AGAGTTTTGGATAACTTTAATTTGCTTTTCTCAATCCTCATACACCACACAATGACAA
CCAAAGTCCTCACTATTTTGACATTTCCGATGGAAATGTCTATATTGAACAAGGAATA
CTTCAATAGATGGTATTCATTGAAGTCATACTACATAGCTACGAATATCCTCGATATA
CCAGTGCTAACTGTTTGTGCGATCACATTCTCAGCAATAATCTACGCAATGAGTGGTC
AGCCATTGGATTGGACCAGATTCAGCATGTTCACTGGCATCAGTCTGTTGGTGGTTTA
TATTTCGCAAAGTCTCGGTTTCATGGTTGGATCAATATTTAATGTGGTGAATGGAACT
TTTGTTGGACCAACAATGCTCGTTCCCATGATGATGTTCTCTGGATTTGGAGTTTCGCT
GAGGGATATTCCAGGATACATGAAATGGGGAACAAATCTCAGTTATCTGCGTTACAG
TCTTGAAGGCTATGTGGCAGCTATCTACGGTTTGAACAGACCAATTCTGCCCTGCACA
AGCTATTACTGCCATTACAAATACCCGAAAAAGTTCATGTCGGAAGTTGCTATGAATG
GAGATCAGTTCTGGTTGGATGTTTATGCTCTTCTTTTCACTTTATTCTTGACAAGGGTA
GCTGCTTACATTCTTCTCAGATGGAGAATAAGGGCGATGAGATAGAACCTTCATGAC
ACACATTAAATCATTAAAATCTTTACATTGCATATTGTACATAACCAACAAATAATAT
TATATTTTTATATTTCGAAGAGCTACTTGTATCTGTAAATAATGAAGACTGAGTTGAA
AAGTTGGAAAAATAGGTTCTCTAGGTTGATATTGAGCCAAAGAAAATTAATGTTATTC
CAACAAAAATGACAAATAAATTTATAATGTTAAGATAAGCTGTTGATATAGTGATTTC
AATAAGGAACTTGGCTGGAATAGC

>NlABCG7
TGTGCTTGGTTGTCTGGAACTAGTTGTTATTTGCATTGCATTATTTGTTGACATTGTGTG
AGAATGAAGATCGATGAAAGTGAGTGCTGTGAGATGAACCAGATTGTTTCTGCTGACA
GGAAGGGCTCGGTGAAGGTGAACATCCAGCCATGCCAGCAGGCGCGCACCCTCACCC
ATCTGCCCAAGCGGCCGCCCGTCGATATCGTCTTCGAGGACCTCACCTACTCCGTCTCC
GAGGGCAGGAAGAACAAAACAAAGAAGATCTTGAAATCTGTAAGCGGCAGACTTTAC
TCTGGCGAACTTACTGCCATTATGGGGCCCTCAGGGGCCGGAAAGTCAACTTTGCTCA
ACATTTTGACTGGTTACAAGACTTCGGGAATGAAGGGTTCAATCAGAATCAACGGAGA
AGAAAGAAATTTGAGCCAATTTGCAAAATTGTCGGCCTACATCATGCAAGACAACCAA
CTTCACGGCAACCTCTATGTTGAAGAAGCAATGCATGTCGCCGCCAATCTCAAATTGGG
CAACGAACATAGCAAAGAGGAAAAATTGGATGTGATCCAGGAAATTCTGGAAACATTG
GGACTACAAGAACACAGGCGCACCCTCACATGCAACCTGTCTGGTGGACAAAAGAAG
CGTCTCTCAATTGCTCTCGAATTGGTCAACAATCCTCCAATCATGTTCTTCGACGAACC
AACTAGTGGTCTGGACAGTTCATCGTGCTTCCAGTGCGTGGCTCTGCTGAAGTCGCTG
TCGAGCGAAGGTCGCACGATAATCTGCACGATCCACCAGCCGAGCGCCCGCCTCTTCG
AGATGTTCGACCACCTGTACACGCTGGCCGACGGCCAATGCGTCTATCAGGGCTCCAC
CGCCCAGCTGGTGCCCTGGCTCAAGACGCTCAACCTCGTCTGTCCCAGCTACCACAAC

CCAGCTTCCTTCATTATTGAAGTATCTTGCGGTGAACACGGCGAAAATGTTCGTAAACT
GATGGCAGCAATCAACAATGGCAAAAATGACATCAGAACTGGCAAGCCGTTCCCGAA
AGTAGATTTGATGGCTCTGAACAACTCCAACATGGAGAAACAGTCCAACCTGAGTCAG
GAAACTGACAATCTGCTGGCCAACGGAGATGCCACTTCGGACTCCAACCAGCCTCTCT
CTGAGGGAAGCAACTGCACCAATAACATGCTGCTAGCTTATGCTACCAATGACATTGCC
AAGGATTCTCAATCGCATTCTGATGGGAAAGGAGGAGTTGTGATTCCAGTGGATTTGG
GTGACAGCGAGAAGGGCAAGTGTGACAACGTTTCCACGAGTTTGTTGGAAACGTCAC
TTCCATTGTCCCAAAAGAGATACGGCACATCGGAATTCAATCAGTTCTGGATCGTTTTG
AAGAGAACTTTGCTCTTCTCACGCAGAGATTGGACACTTATGTACTTGAGGTTGTTTGC
TCACATTCTGGTTGGCTTTTTGATTGGCGCTCTCTACTACGACATTGGAAATGACGGAG
CCAAAGTGCTCAGTAACCTTGGATTCTTGTTCTTCAACATGCTCTTCCTTATGTACACAT
CGATGACCATCACTATTCTCTCCTTTCCTCTTGAGATGCCTGTGCTGATAAAAGAAAAC
TTTAATAGATGGTACTCGCTACGATCCTACTATCTCGCCATAACTGTTTCTGACATACCAT
TCCAGGCTGTATTCTGCGTACTGTATGTGTCAATTGTGTACTACTTGACATCGCAGCCGC
AAGACCTATCTCGTTTTGGAATGTTCTTGGGAGCTTGTCTGCTCATCTCGTTTGTCGCG
CAGAGTGTTGGACTGGTCGTGGGAGCCGCTATGAATGTTCAGAACGGTGTATTTTTGG
CTCCTGTGATGTCGGTGCCCTTCCTACTCTTCTCCGGATTCTTTGTGAGTTTCGACGCTA
TACCGGTCTATTTGAGATGGATAACATATCTCAGTTACATCAGATACGGATTCGAGGGAA
CTGCACTTGCTACCTACAGTTTCAACCGAACAAATCTTAAATGTTTCCAGGTATACTGC
CACTTCAAGGACCCCAACACCACACTGGAAGAGCTCGACATGAAATCTGCCAGCTTC
GAACTAGACATCATTGCTTTAATTGTAATATTCTTTTTCTTGAGAATCTCAGCATACTTAT
TCCTTAGGTGGAAACTGATGTCGTCTCGTTAATTTCATTCCAATATAGCATTAGACTAAA
TAATATTTTTACTGGAATCCAGTGCCTTAATGCTCA

>NlABCG8

TATCACACTGCGATGCTCTCATTGTAAACAAAAGCGTAGTCGTTCAGCTAATAGCTGT
AATCGTGTGCAGTGCTTTTCGTTGCGAAGATATTGACAAAGTGCCATGGTGTTCATTC
CTAGTGTTGCTCAGAAGTGAATCGATAACTGCTAGATTTTGAAAGAGTGAATCAGTCT
ACAATGGAGGGTATCAACTCGAATAAAACTAAACACGACATGGAGCTATACAATAAC
AATGACTACGATCAGTTCAGCTTCCCCAAACGACCCACAGTGGATATTAATTTTCAGG
ACATCACGTATACTGTGAATACAATGACAGCAAAAAAGGAGATTCTGCACGGTGTGA
GTGGAGAATTCAGGTCTGGAGAGTTGACAGCCATCATGGGGCCTTCGGGTGCGGGAA
AGAGCACCCTACTCAACATTCTGGCTGGATTTACGTTGAAAGGCTGCCAAGGAAGTA
TATGCATCAATGGTATTAACAGGAAGAGTCGAATCGAACAATTCCTGAAGATGTCCT
GCTACATTACACAAGATGACGAACTGAGGCCTCTATTGACTGTCAGAGAAGCTATGA
TGCTAGCAGCGCATTTAAAACTCGGTTTCACTCGATCCAACCCTGACAAGTCAAATCT
GGTATCATATATATTGGGTCTTTTGGGACTAAAAAAACATGAGAATACTAGGACATCT
CGATTATCTGGCGGACAACGCAAAAGGTTATCAATTGCTCTCGAGCTGCTCACCAACC
CTCCAATCCTGTTCCTTGATGAGCCAACAACGGGACTAGACAGTGTGTCAACCACGTC

ATGTGTTTCGCTGCTGAAGAATCTGGCGGCTGAGGGACACACGATCGTCTGCACAAT
CCACCAGCCGACTGCCTCGATATTCGAGATGTTTGACCACCTGTATGCAATCGCAGAC
GGCGACTGCATCTATCAAGGCTCCTGCAGCAATCTGCTGCCGTTCCTGTCCTCATTAA
GTCTGCATTGTCCCAAATACCACAATCCTGCAGACTTTTTAATCGAAGTGGCAATTGG
TGAATACGACACAAATGTGAAGACGATAGCTGCAGCGGCAGCCAAGCATGGTCGCCA
AGAGTCCACACCTTATTCCGAAGAAATAATCAAAGATGAAAATGGTCTGCCAGTTAG
AGTATTGCAAACCAAAAAGTCGCCAGCTTTTGACATAATTGAGTACACTTCATGTTTA
GCAGAGCCGGCTCCGTTATGGTATCAAGTATTTCATCTTCTCCATAGAAACATAATTA
TCACACGTAGGTCAAAGTTGCCGCTTGCCTTACGAATTTTTATGCACTTTGTGATCTCA
GTAATGTTTGGAATCATTTACAATAATGTTGGCAACAACGCAAACTCTGTATTTGGAA
ATTACATTTACGTTTATGGAACGAACTTGTTTTTACATTATACCGGACAAATGGCAGT
TACTTTGTCGTTTCCACTGGAGTTCAAAGTGCTGAGGAGAGAGCATTTCAATAGATGG
TATTCTCTGATTCCTTACTGCATAGCGACGCTACTAATTGAGATACCGTTTCAGATTGT
TTGTGTTGTCGTGTACTTGGTGCCCAGCTACCTGTTGACTGGACAGCCCCTGGAGTGG
GTCAGGTTCTTGATGTTTCTAATGTTCACGGTTGCAGTTTGTCTGACTGCGCAAGCGTG
TGGATTTTTGGTTGGAGCCACAACGCCAGTAACGTTGGCTGTCTTCATTGGACCAGTG
ATCACTGTATTCCTATCAGTGTTCGGATTCGCAATGAAGTACAGCGACATTCCGTCCT
ACCTGCGGGTCTTCTATCACATATCATACTTCCGATCGTCCTTTCAAGGATCACTGAT
GAGCTTGTACGGTAACAACCGGTCCTACCTGCCGTGTCTGAAGAACGGGTTTCACGG
CCGTAACGGCTACTGCCACTACACGCATCCGACAAAGTTCCTCAGGGAAATGGAGTT
CGAAGAACCAAACCCTGTGTTCGATGTCAGTTACATTGTTTCTGTTTGTTTACTGGTGT
ACACATGTACAGCGACTGCTATCTGGTATAGATTGAACAAAAGGTGATAGATTTCTA
GCTCTTTGGTAAATACATGTTCAGCGACTGCTATTTGGTATAGATTGAACAAAAAGTG
ATAGATTTCTAGCTCTTTGGTAAATACATGTTCAGCGACTGCCTCT
>NlABCG9
ACTGACCGCTAGTGTGTAGAGCGGTCGACTTTTCAATCAAATCGTATTTCATGTGCCG
TTGAAATCGAAAAATGGACGTGGAATTCGAGGATTTGACAGTCAGAGTGAATAGCAG
TTGGTTTAATAGAGATTCTGGAAGAAGAATTTTGAAAGGAGTGAGTGGCAAATTCAA
AGCTGGTCAGCTATCTGCCATACTTGGACCTTCAGGCGCCGGAAAAAGCTCTCTACTC
AATGCAATATCTGGTTACCGATCTCAAGGCGTGAGTGGCCGTTTGAGACTGAACGGA
GTGGCAAGAGATGAAGCCAGGTTCAGAAAGATGTCGTGCTATATCCAGCAGGAGGAC
CTTTTGCAGCCAATGCTCACTCTCCAGGAAGTCATGAACTTTGCAGCCTTACTCAAAC
TTCCGCCAGGAACTGGATACAAGCAGAGAAGAGTTGTGGTGAATGATATTCAAGGAA
TTCTGGGCTTGACTGAATGTAGACATACAAGAACTGAGGCGCTGTCTGGTGGCCAGA
AAAAGAGACTATCTATAGCCCTAGAGCTGATCAACAACCCTCCAGTCTTATTTCTAGA
CGAGCCTACGAGTGGCTTGGACAATGTGTCGACGTCGTACACTCTGCGTCTGCTGCGA
ACCCTGGCCCACCAGGGCAGAACGATAGTGTGCACCATACATCAGCCAAGTGCCAGC
CTCTTCCAGATGTTCGACCACGTGTATGTGCTGGCCTCGGGGCTCTGTGTGTACCAGG
GCGTAACCGGCGAGCTCGTTCCCTTCCTATCCTCGGTCGGTCTACACTGTCCCAGGCA

TTACAACCCGGCTGACTTCGTAATTGAAATGACCGATGGAGATGACGAGGATAATAT
CAAAAGATTATCTTCAGCGATAAAAAATGGAAAAGTTGTCCAACTGACGTCTGCTGA
TGCCAAAAAGTCAATTCCAGATTTTTCAAATCTACCAATAGAAGGGCTACCACTTGAA
GAGAAAATAGTTGCAGTCACTGGTTCTGGAGAGAAATACGTAGACATGGACAACGGC
ATCTGTCTCACTTGCCGGGCAGACTCATCATCTTGGTTAGAATTCTGTACGCTTTTCAG
AAGGATGTTCCTTCAAATTATGAGAAATAAGACTGGTCTAAAAATCCAATTCTACCAT
CACTTGGTGTGTAGTCTGGCTGTGGGCATTGTATTCTGGGGCAAGGCACGCGACGGA
AATCAGTTCTTCAACCACATGAAGTTCTGCATGGGGATCATTCTATTCCACGCCTACA
CTCAGTGCATGGTACCTGTTCTTACCTTTCCATTCGAAGTGAAACTTTTGAAGAAAGA
ACACTTCAACCGATGGTATCGACTCACGCCCTACTACATGGCTCTACAACTCTCCAAA
GTTCCAACCATGACGATATTCAGCCAGTTGTTCCTGACAATAGTGTACGTGATGTCAG
GTCTACCAATGGAGTTCTACAGGTTTTTCGTTTTCTCAGTGGTTGGCGTCATGACTGCA
TTTGTGGCTGAGGGATGGGGTCTTGCCATTGGATCTGTATTCAATGTCACTAACGGCA
GCGCAGTGGGACCGATGACAATTGCGCCGTTTCTCGGATTCGCGATCTACGGCTTCGA
CTTTGCCCGAGACATTCCGGCCTGGTTCATGCCCATCCTCAAGCTGAGCTTCCTCAGA
TCGGGCGTCATCGCTCTCATCATCGTGGTGTTCGGCATGAACAGGGGTTTCCTCGACT
GCAACCACGAGATGTACTGCCACTTCAAGAATCCTCGCATCATCATCTACTACCTGGA
CATAGAGGGAGTGTCGCCCTGGCAGGAGATACTCGGCATGTTTGCCATGCTCCTCTTC
TTCAGAATTGTCTGCTTTTTTGGACTAAAATGGCGGCTCAGAACTTGAT

>NlABCG10
GTGTTTCTGGACAAATGTTCAGTTTTTTCGAGATTTGAGTGTTTTCAAGTGGGAATAAT
GATTGGCAACGACTATAGTTTGGAGCTGTGTAATATTTTTCACACTGGACAGGTTGAG
CCAGGCTCATGTTTGCAGAGAATATTTGGCAGCGTGCAGACTGGCCTGATTCTGAAA
GATGTTTCTCTCGAAGTGAGAGCGGGAGAGGTCCTCGCAGTTCTTGGATCGAAAGGA
AGTGGTAAACGAGCTCTTCTGGAAGTGATATCTAGGAGAAGTCGTGGTCCCACCAGA
GGACAAATCCTCCTCGATGGAGCACCAATGACCTTGAGTTTGTACCAGAAGAATTGC
GGCTACGTCAGTCACAGAGTTGACCTAATTCCGTCTCTAAATGTGGAGCAAACTCTAC
ACTATGCGGCCAACCTCACTATTGGATCACAGGTATCACGGTACGTGAGAAGCTCTC
GAGTTCGTCAAGTGTTGGCAGATTTGGCTTTGAGTCAAGTGGCTCGGCGAAGTGTGGC
AAGTCTCACACTCAGTGAATACAGGCGCCTTGCCATTGGTATACAACTCGTCAAGGAT
CCTGTTCTGCTGCTATTGGACGAGCCGACCGCTAACCTTGACCCTCTGTCGACCTACC
TGATAGTGTCGATGCTATCCTCACACGCCAGACGGAGGGGGAGGGCGGTGGTGTTGA
CCATGGAGAAGCCGCGATCCGACGTGTTCCCCTTCCTCGACAGAGCCGCCTACCTCTG
TTTGGGGGATCTCGTCTACGCGGGACCCACTCGCCTCATGCTCGAGTACTTCAGGGCC
ATCGGCTTCCCCTGTCCTGATCTCGAGAATCCTCTCATGTATTACTTATGCTTATCAAC
AGTGGACCGACGTTCGAGAGAACGCTTCATCGAGTCCAACACCCAGATCATAGCTCT
TGTGGAGAAGTTCAAACTGGAAGGAGGTCCCTACAGGAAATCATCAGCCGGAGCGG
GGGGAGCGGGGCACGTTCTTCTGGGTGCGGGGGAGTCACCCCCTTCCCATAAAATGC

CCCTCACCACCCTCGGCAAGCCAGGGGCTCTGCAACTTGGTTTCACACTCTATCAACG
CCTGCTAGCATCCACATTCAATCTGTCCTCGATCGCAGCCCAAAACCTCTTTCTACAC
CTGGCACTGTTTCCCCTCATCTGCACAGTCCTCTGGTTCTTCTACCGGGACATCAAGC
ACCAGGACGGACCTTTCACGTTCCAATCACTCAACGGATTCCTGCTCAACTGCCTCAT
CACTTCCAGTGCATGCGCAGTTGTCAAAACTGCCTGTACATTCCCCATACACCGGACC
CGATTTTACCAGGAGGCTCACGAAGGACTATATTCTGGCCCATTATTTTTGCTTAGTTT
TAACCTATATTCCTTACCGTTTTCGATATTAACTGTAGCTATCGGGTCAAGGATATTGT
TTGAAGCTACAGGTCTAACTTCTAGTGTAGACTGGTTCTTATTTGCGGCGATACTCTG
CTCCTGTTATCTACTAGCGGAGCAACAAACGATCGCTCTATTAATGGTGATTAAAGGA
TCATTCATCGCCGCGATAACCAGCTTATACCTGGGCACAATATTCATCATCCTCAGTA
GTGGAGCTTTGAGATCGTATGCTAGTCTACCAGAATGGTTGCTCTACCTAACCTACGC
GTCACAAACGCGCTACTCCAGCGCGTTTTTGTCGCGTCAGCTTTTCGGGTCTGTTTAC
ACCGCGTTGCCCGCTAACTGTACAGCGCGATTTCCCGTCAACGACGCGTTTCTATGTC
GTTACAAGGACAGTACTGCGTACCTGGCTGAACGCTTCGGTCGAGGCAGTTCAGTTTT
CAACATAAATGACATGTTAGATTCCGATTTCAATCTCAGTTTATCGTATGCTTTTCCAG
TCGGTTTTGTTTTGTTGAACTGTATCCTGTATCTGATACCTTTACCCTCGTTTATAAAA
GCTAAGTTTAGGGATTGAGGGTTTTTTTTGTTGAACTCCAGGTGTATATATACATAGA
GGTTGTGATACCAATTTTAATTTTGGAGGTAGACTCAATTTTATTCCTTCACGAAATAT
TGATACAAATTTATGAAATTCATTTAAACATTTAACAGTTTCCTACATAGGAGGTAGT
ATTTTTTTTAATCCTCTGGTTACACTGTACTTCATTGTAGCCTGGAGTTATAGGAACTA
TAACACATTGTTAATAGTGTACCATACAGTTATAGATATAGCCTAATACATGCAATTC
AACACATTTTATTGTGTTCAATGCCGTTAGTAAGCGCTTATTATAGTGTGCTAATGCTA
AAAAGGAGGTGGATGAGTATAGCA

>NlABCG11
ATGACGGAGGAACGTGCTGTTTTACTTCACTTGCCTCCCAGCAAACCTATTCACATAT
CGTTCACTGACATCACTCTCACCGTCGACTTGGGCACTGTACGAAAAACGAAAAAGC
AAGTGCTGAAAGGTCTCGCTGGGAGTTTCAACTCAGGAGAGCTGACGGCCATAATGG
GTCCATCGGGTGCTGGGAAATCATCGCTACTCAACATCCTAACCGGCTTTCAGAAACA
AGGCATGACGGGTACAATAACAACGAGCGGAGCTGGAAAAATAGAAAACTATTTCA
AAGATGGCGTCAACACAAAACAGTCCTGTTACATAATGCAAGATGATCAACTGAACC
CTCTGTTCTCAGTTTTTGAAATAATGTCGATGGCTGCTGATTTGAAATTGAGTCCAGCT
ATATCACAGAAATCTAAAATACTTATTATTGATGATATTTTGGAGACAATTGGTCTGA
TGGGTTGTAAGTACACAAGGTGTGGCAGATTATCGGGAGGCCAGAAGAAAAGGTTGT
CCATAGCTCTGGAACTTGTTGACAATCCTCCGATCATGTTTCTAGATGAACCAACGAC
AGGATTGGACAGCTCAAGCACGGTTCAACTGGTTTCTCTACTAAAATGTTTGGCAAGA
GGCGGAAGAAATATAATTTGCACAATCCATCAGCCGAGCGCAACAATCTTTGAAATG
TTCGACCATGTTTACCTCATCAATGGCGGACGATGTGTGTACCAGGGCTCTAGCATCA
ACTTGGTCAAATTTCTGCAGTCAATCAACATTCCTTGTCCCAAGTACCATAATCCTGC

CGATTTTGTAATGGACGTGATAAGTGGAGAATTCGGTGACCATACTGACAGGATGAT
AGATGCATCTCAGAACAGCAATTGGAGGGCTCCGCCACCTGTTATCAGAGCACAATT
AAAAAAACGAACTTCGGATGATATTGAAAAAGTGAAAATGATGGGCTTTCCGGTAAC
ACCACCAGAAATTCTAAGGTTATGGGTCCTCATCAACCGATGCATTATCCAACTGTAT
AGGGATTGGACTGTAACACACTTGAAAATGATAATGCACTTCCTTGTCGGCGTAGTCA
TGGGACTCATTTTCAACAAGTGTGGCAATGATGGTAGTCTTAGTGTAAATAACGTAGG
CTTCTTCCTGTGCACAAACGTATATCTGAGCTACACCTCGATAATGCCAGCGATTTTG
AAATTTCCCTCAGAGCTCCACATACTGAAAAAGGAGCAGTTCAACAACTGGTATAAG
CTCTCCACCTACTACATTGCATTCCTTCTCACTAATATTCCTGTACAGATGATGCTATG
CACAGTGTATGTGTCAGTCTCGTACTACCTGACGCATCAGATCCAAGAGTGGCCGCGT
TTCGCCATGTTCTTGGCTGTCAATCAATTCTCAGTCATCATATCAGAGTTCATCGGCCT
AGCACTTGGAACAACTATAAATCCTGTGAACGGACTTTTCACAGGCTCCGTCCTATTC
TGTTTCATGCTACTATTCGGGGGCTTTCTCGCTCTCTACAAGCACATGACACTACCACT
CTACTTAATCTCGTTTCTAAGCTACATGCGTTACACCATGGAGGGGATGGTGCTCTCA
ACATACGGCTTCCAACGTCCACTACTCGACTGTCCAAAGAACTACTGTCACTACAGAA
TACCCTCCGTCGTACTGGACGAAATCGACATGAAAGAAGACCATTATTGGTGGGACG
TGATTATTCTAATCAGTATGTGCGTTTTCTTCTCGTTTCATGCGTATGTCACCCTGAAA
CGACGCGTTATGCATAGGTAAAGAGTATTGAAAAATGGTTTCTGCGAGAGAGGTAAG
AATCGATTCTATTCTACAATCACATCTATAGTATTTGAACGCTGCTGTTTTGAAGATTC
TGAGAGTAAACAAAGACTGAATTTCAAAGAGTGTGTGAGAATGGACCTGTTGAATGG

>NlABCG12
CCTACTATGCTGTAGTGACTACAATAATTTTTCAAAATGTGTGCCAAACGTCATGGACA
AGTTACCATCGATTTCGAGAATTTGTCTTACAGTGTACCTGAGACAAATAAAAAATATC
CTGACCGTGTTCTTCTACACAACGTTAGTGGACACTTCCGTCCGAAGCATTTAGTGGCC
TTAATTGGTCCATCAGGAGCTGGTAAAACTACCCTTCTAAATGTAATATCTGGTTCAAA
GGCAGTAGACAATTCGCGAGTGTCTGGGAAGATACTCGTAAATGGAAAAGATAGAAAT
TTGCAAAAGTTTAAGAAACAATCCTGTTATATTACACAGGAATGGTCTCTACTGAACCA
GCTGACTGTAGAAGAGACTTTGGAAATTGCCGCTAGATTCAAGCTGCCAAGCAATATC
AGTGAAATTGATCGGAAAAGTTCGATCAATGAAGTTGTTGAAATTTTGAGACTGAATG
GATCTAGGAACACTCTGGTAAAAAATCTTTCAAATGGTCAGAAGAAACGAATTTCAAT
TGGTGTTGAACTTATGAATAATCCACCTGTTTTGTTCGTGGATGAGCCAACAAGTGGGT
TAGACAGTTCATCAGCTCTGCAAGTGGTTAATCACCTTCAGAGTCTGGCATTGGACGG
CCGAACTGTGATAGTAGTGATACATCAGCCGAGCTCCAAAGTGTTCCAGCTGTTCCATG
ATGTCTACCTGCTGTCTGATGGTGAATGCCTGTACAATGGTCCCTCCGAGCACTTGGTT
GCTGCCCTCTCGTCAGCTGGCTTCAATTGTCCACAGTATTACAGCAAGTCTGATTTCGC
GATCGAAGTCGCCAGCGCAGAGGTAGAAGGAGATGTAAGATTATTAAAGATGGAAACA
AAGAAACGCTATGAAATGAAAGAAGGAAAATTTTATGAGAATGAAATACCTGCAAAGC
GAATTTCAGAAGCGAAGAATGCAAATGAGCACACAACAATGATGATCGGTGAAGATG

ACGATGATGATTCCTATAATAACTCTTTGAGAGGTTATCCAGTTTCAAAACTTCAACAGT
TCTGGATACTCTTCAAACGATGCACTCTTTGTACGAATAGAGATATGTATTTATCTCGAA
CTCGATTAATTACGCACATTTTGGTTGGCATCATGCTCGGCTGTCTCTTCTACAACTTTG
GAAATGACGCCGATAAAGTAATAGGAAACTATTCCTTCTATTTCTTCGCCGTATTATTCA
TCTACTTCAGCAGTACTATGCCGGCCATAATGACATTTCCGGTTGAAGCCAACGTATTCC
TTAGAGAGCATTCAAACAATTGGTACTCTCTTACCGTCTATTTCTTTGCAAAAGTATTAG
CGGATCTACCCTTACAGATAATCTGTCCGACTTTATTTTTGGTGATTGGTTACTCCATGA
CAGGCCAGCCAATGGAATTACAGAGATTCTCCATGATTTGGTTTGTGATGGTTCTACTG
TCCATCCTTGGCCAGTCGTTCGGCAATGCGGCTGGTGCTCTGCTCAATGTCGAGCTGG
GCATCTTCATTGTGCCGTCGATTGCGATGCCTCTGACGCTGCTGTCGGGTTTCTTCCTG
CAGCCGAAGGACCTGTCGGCGACGGTGAAGACGCTTAGTTGCGGCAGCTACTTCAAG
TACGCATTCGAAGCGATCGCCGTGTCGGCCTTCGGCTACGACCGTGGTCGGCTGCCCT
GCTCGCAGCCCTACTGCCACTACCGCAGCCCGGCCAAGTTCCTCGCCGACATCGGCAT
CGATGACTATCACTACCTGCAACGCGTCACCGTCGTCCTCGTTTGGGTCCTAGTCACGC
AAATGGCGCTCTACTGCACGCTCACCATCAAGGTCTACCAGGTCAAGATCAACACTGC
TCTGCGCAGGCTCATGAGATGAGCTTCAACTGCACTCTAGTGGTT

>NlABCG13

GCGATGATGACTCGTGACATTTGATTTGAGCCGGCTAACCTCGTTTCGTGTGAGTTAG
CGCGCGGTCCACTCTGCACAACGCAACGTTCAATTACACACCATCTCCTCCTCGAGGG
ACATGATAATTTCCAAGGATCTATTCCTGCTGGGTGACCAAGACTATCTGCGTCTTCC
CAAGGACGAGAAGCGGACCATGGTGCGCCCGACCTCGGCCAAAACTCAAAGGGTGG
AATCGCATTTACCATGTCTTCAGCACCATCACTTGCCTCACAGACCACTGGAGCTTGT
CTTCACCAACGTATCCTACGTTGTGGACAAAAAAGCTATCCTGAAGGACATCAGTGG
TGTTGTCAAACCAGGAGAGCTGTTGGCTGTCATGGGCCCTTCAGGCTGCGGCAAAAC
GACCCTTCTAAATTGCCTGGCCGGCCGATTAAAGTTAGATTCCGGAAATATTCGTCTG
AACAAAGAACGCCTTAACAAGCGCTGGAAAAGGCGGATATGCTACGTTTTGCAGCAA
GATATTTTCTTCCCTGATCTCACACTTCGTCAGACCCTAGAGTATGCAGCGATGTTAC
GGTTACCTGATTCACTATCTCACGCCCAAAAAATGCAATACGTTGATCACATAATTGA
TGTTTTGGATCTTACGAATTGCCAAGAAACAATTATAGGAGATTACATAAAAAGAGG
ACTGTCCGGGGGAGAGAAGAAGCGAGCCAACATTGCCTGTGAACTACTAACCAATCC
CTCATTGATGCTCCTCGATGAGCCGACTTCAGGACTGGATTCACATTCTGCTTACAAT
TTAATGTTGTCATTAAAAAAGTATGCGGAAAAAGAAGGAAAAACAGTGGTTGTCACT
GTTCATCAACCCTCTTCTCAAATATTCCACATGTTCGACAGACTATTATTACTTTGCAA
TGGAGAGACTGCCTATTTTGGGGACGTAAATAAAGTTGTTGACTTTTTCAACAACGTT
GGACTGACGATGATGCCTCATTACAACCCTGCTGACTTTATTTTGGAGCAAGTAAAAG
GAAGTGAAGAAATGAAAGAAAAAATCATCACAGCGGCGAGAGAAGCCAGATTTCGA
CCCAACTATCCCCAAGAACTGATGCCGGAATATTTCAACCAATCAATGTATCTAAATA
ACTATCACGAAAGTCATTTACATTCGAATGGTCACATAGGAGGAGTACGTTGTCAAT
GCCAGCGAGAATTATGGAACAATTCAAGGCACTCGACATCACAGAATCTACCCTCTT
CCGAAATGTGTGTCCCGGTCGCCGTTGCGATAGGCGAAACAACCGAATCGCAGGGAC
ATGTTTACACAACTATTGCAGTGAAAGAAGAAGAGGGTAAAACCCTGTGGCAGGATA

CAGCCAGTCACGCGTCTTCCTCGGTTAGCAGTTCTGACGACGATGTGTCGTGGCAGTG
GCCCACTTGTTTCTGGACTCAATTCAAAGTACTGAGCAGAAGAAATTTCCAAGAAGC
AAGACCCAGAATGCTTTCAACTTTGAACTGGGTACAAACGGTAGCTTTAGGAGTGAT
GGCTGGACTTCTCTGGTTTCAACTGGAGAGGAAAGAGGAGTCACTACATGACATCCA
GGGATGGATGTTCTTCTCAACGACGTATTGGATGTTGTTCGCTCATTTTGGAGCCCTAT
CGTCATTTCCACCTGAAAGAGAAGTGATCAACAAAGAACGTCTGTCTGGAGCATATC
GACTGTCGGCCTATTACCTGGCGAAAATGGTGGGCGAATTGCCGTTGACCATCACGCT
GCCAGCAGTCTATCACATCATATCCTACCCAATGCTTGGCTTCCACAGTCCTACCGTG
TTTGCCACACTGCTGGGATTCTTGCTTCTCAACACTATTGTAGCTCAGAGTGTTGGATT
CTTTGTCGGCGCATGCTGCATGGACATGCAGGTGTCGATAACGATCAGTGCCCTCTAC
ACGCTGGCCACGCAACTGTTTGGCGGTTACCTGGCCACCAACATCCCGCCCTGGCTCA
AGTGGATGCAGTATTTGTCCATGGTCCATTACGCCTATCAGAATATGCAGATTGTCGA
GTTCAGCGAAGGAGAGTGGATCAAATGCGCGCCGCAATCGAAGTTCGACGTGTGCCT
CAACAACTCGACGACGCACATCCCCGTGGCGTCAATCCTGGAGGTGCAGGGTGCCAG
CCTGCCGCTCTGGGCCAACACCCTCGTGCTGCTCCTCTTCCTGCTCATATTCCGCGTGC
TCGGCTACATCGTGCTCAGATACTTCCGTCGACCCAAGTGAACTCGCCAACA
